# Evolutionary Changes in Crassulacean Acid Metabolism (CAM) and Related Traits During the Diversification of *Aichryson* (Crassulaceae) on the Macaronesian Islands

**DOI:** 10.1002/ece3.72864

**Published:** 2026-01-02

**Authors:** Jessica A. Berasategui, Thibaud F. E. Messerschmid, Stefan Abrahamczyk, Ángel Bañares‐Baudet, Nadine Bobon, Gudrun Kadereit

**Affiliations:** ^1^ Prinzessin Therese von Bayern‐Lehrstuhl für Systematik, Biodiversität & Evolution der Pflanzen, Ludwig‐Maximilians‐Universität München München Germany; ^2^ Botanischer Garten München‐Nymphenburg, Staatliche Naturwissenschaftliche Sammlungen Bayerns München Germany; ^3^ Botanischer Garten der Universität Osnabrück Osnabrück Germany; ^4^ Departamento de Botánica, Ecología y Fisiología Vegetal Universidad de La Laguna Tenerife Spain; ^5^ Institut für Entwicklungsbiologie und Neurobiologie Johannes Gutenberg‐Universität Mainz Mainz Germany

**Keywords:** δ^13^C, abiotic stress, C_3_‐CAM, CAM inducibility, climate‐chamber experiment, Crassulaceae, diurnal acid rhythm, minimum leaf conductance, molecular phylogeny, succulence

## Abstract

Crassulacean acid metabolism (CAM) is a highly plastic photosynthetic pathway with ecological and evolutionary significance, ranging from weak inducible to strong obligate forms. While most Crassulaceae taxa may be capable of performing CAM, the Macaronesian genus *Aichryson* has not traditionally been associated with CAM. We integrate phylogenetic, physiological, isotopic, anatomical and bioclimatic data to investigate the distribution, plasticity and evolutionary history of CAM and related traits in *Aichryson*. Our study includes all 15 accepted species, combining over 1100 occurrence records, carbon isotope (δ^13^C) data, nocturnal acid titration and a CAM performance experiment under temperature and drought gradients. Multivariate analyses of bioclimatic variables show clear ecological differentiation among the Azores, Madeira and Canary Islands, with life form strongly associated with climatic niche. Annual species are generally restricted to cooler, wetter climates, while the perennial *A. tortuosum* lineage, endemic to the arid eastern Canaries, exhibits increased succulence, lower minimum leaf conductance and higher CAM performance. Ancestral state reconstruction of δ^13^C data suggests that the ancestor of *Aichryson* possessed a predominantly C_3_ physiology with low‐level CAM capacity, from which independent shifts towards stronger CAM expression or reversions to predominant C_3_ photosynthesis occurred in response to local climatic conditions. Our CAM performance experiment revealed pronounced interspecific differences in nocturnal acid accumulation and plasticity. Some annuals, such as *A. bollei* , exhibited high CAM inducibility under stress, while others, like *A. dumosum,* maintained low ΔH^+^ across treatments, likely reflecting relaxed selection in mesic habitats. These physiological traits align with environmental niche and life history, supporting two main strategies: fast‐growing annuals with flexible CAM and slow‐growing perennials with more constitutive CAM and investment in leaf longevity, cuticular properties and water storage. These findings support a ‘CAM continuum’ and highlight the roles of ecological differentiation and climatic filtering in shaping CAM evolution. *Aichryson* emerges as a model system for understanding CAM plasticity and the interplay between photosynthetic pathways, life history and insular biogeography.

## Graphical abstract

We explore evolutionary changes in Crassulacean acid metabolism (CAM) and associated traits during the diversification of *Aichryson* (Crassulaceae) across the Macaronesian Islands. By combining field data, physiological measurements and phylogenetic analysis, we identify shifts in photosynthetic strategy linked to habitat transitions. Our findings highlight the role of CAM evolution in island adaptation and lineage diversification.

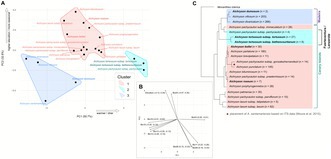

## 1 Introduction

Introduced as a photosynthetic curiosity in 1947 at a meeting of the Society for Experimental Biology (Black and Osmond 2003), Crassulacean acid metabolism (CAM) quickly gained recognition for its broader ecological and environmental implications than previously envisioned (Osmond 1978). To date, CAM has been discovered in at least 38 plant families and approximately 16,800 species (Silvera et al. 2010; Gilman et al. 2023), and different types of CAM photosynthesis have been proposed (reviewed in Winter 2019; see also Edwards 2023). The unifying features of all CAM types are the nocturnal fixation of CO_2_ by phosphoenolpyruvate carboxylase (PEPC), the accumulation of the resulting malate in the vacuole and the gradual consumption of the stored carbon during the day to fuel Rubisco, while the stomata may or may not be closed during part of the day. The CO_2_ comes either from nocturnal gas exchange requiring inverse stomatal opening, in which case acid accumulation can be high (constitutive, obligate and facultative CAM), or from respiration, in which case acid accumulation is always low (CAM cycling, CAM idling). Early reviews of the progress of CAM research have acknowledged that this pathway is likely to be highly plastic and inducible (Ranson and Thomas 1960). With more experimental studies on the physiology and biochemistry of individual species, it became clear that there is a wide range of species‐specific environmental triggers of CAM, such as temperature, drought, salinity, light intensity, photoperiod and carbon deficiency, and that there is a continuum of CAM phenotypes from plant species that only occasionally perform CAM under stress and otherwise engage in C_3_ photosynthesis to obligate CAM plants that perform CAM regardless of environmental cues (Cushman 2001; Dodd et al. 2002; Winter et al. 2015). Furthermore, the observed CAM phenotype depends on the developmental stage of the leaf and whole plant or on the stress memory of the plant (e.g., Pintó‐Marijuan et al. 2017). This enormous variation and plasticity have been recognised in several reviews on CAM photosynthesis as the ‘CAM continuum’ (e.g., Silvera et al. 2010) and have complicated the definition of a ‘typical’ or ‘true’ CAM plant. In the past, the δ^13^C value has often been used to infer the amount of CAM activity during the lifetime of an organ, but it has its limitations, especially when it comes to distinguishing between facultative CAM and C_3_ photosynthesis (Hancock et al. 2019; Messerschmid et al. 2021).

Bräutigam et al. (2017) proposed a model of CAM evolution in which selection acts on pre‐existing, low‐level nocturnal metabolic fluxes already present in ancestral C_3_ lineages. According to this view, CAM evolved not through a complete rewiring of metabolism, but through quantitative shifts in flux, specifically, an upregulation of carbon fixation and organic acid storage pathways that may already occur to a minor extent in some C_3_ species. However, this model has been the subject of ongoing discussion within the CAM research community, particularly regarding whether such fluxes genuinely represent precursors to CAM or are incidental (Edwards 2023). Alternative perspectives emphasise that CAM evolution may have involved more substantial regulatory and anatomical innovations, including shifts in stomatal behaviour, vacuolar storage capacity and diel gene expression control. The repeated and independent origins of CAM across many plant lineages support its evolutionary accessibility but also point to a variety of biochemical and regulatory fine‐tunings adapted to different ecological contexts. It has been suggested that evolutionarily early or intermediate CAM forms may not have relied on fully inverted stomatal rhythms but instead exhibited partial nocturnal opening, especially during dusk and dawn, combined with limited daytime closure in response to water deficit (Winter and Holtum 2014). In stronger CAM plants, however, daytime stomatal closure is mainly regulated by elevated intercellular CO_2_ concentrations resulting from malate decarboxylation rather than water availability per se (Dodd et al. 2002).

Given this framework, one might expect weak or facultative CAM to be more widespread in nature than currently documented. Yet such forms are difficult to detect, especially when CO_2_ assimilation remains dominated by the C_3_ pathway. Moreover, reversions from weak CAM to C_3_ photosynthesis appear possible under relaxed environmental selection (Hancock et al. 2019). Interestingly, even strong CAM species seem to retain residual C_3_ function. In *Kalanchoe*, for example, PEPC1 knockouts generated by Boxall et al. (2020) showed impaired nocturnal malate accumulation and CAM deficiency yet remained viable under well‐watered conditions by relying on residual C_3_ photosynthesis. This highlights the metabolic flexibility and reversibility of CAM traits.

To understand the initial evolution of this complex pathway, more case studies are needed that combine phylogenetic and experimental approaches and examine CAM performance and CAM‐related traits. One of the few studies examining C_3_ and C_3_‐CAM species (i.e., species that primarily perform C_3_ but engage in CAM to a lesser degree) in a phylogenetic context is the work of Hancock et al. (2019) on *Calandrinia* (Montiaceae), a genus of approximately 70 species of mostly small, leaf‐succulent, annual herbs endemic to Australia. Although widespread in arid regions of Australia, none of the *Calandrinia* species seems to engage in strong CAM. The authors hypothesise that the annual life history and rapid completion of reproduction select against strong CAM, which typically involves an investment in plant and organ longevity with slower growth rates. Instead, the study suggests that CAM is a labile trait with frequent reversals to C_3_ photosynthesis. At the same time, Hancock et al. (2019) highlight the difficulties of using δ^13^C values to detect CAM lineages among lineages with predominant C_3_ photosynthesis. A similar conclusion regarding the evolutionary lability and environmental inducibility of CAM is drawn by Winter and Holtum (2024), who emphasise that facultative CAM represents a highly plastic and environmentally regulated photosynthetic mode. It is likely more widespread in nature than currently documented, but also more difficult to detect.

Niechayev et al. (2019) highlight the need to study CAM‐associated traits that play a crucial role in the evolution of the CAM syndrome as a whole. Such co‐adaptive traits include, for example, succulence, extent of intercellular air spaces, chemical composition of the cuticle and its transpiration barrier properties, and the distribution, density and responsiveness of stomata in photosynthetically active tissues. Succulence has often been studied in relation to CAM (e.g., Silvera et al. 2005; Herrera 2020), based on the simple hypothesis that CAM activity is optimised when the vacuole size, i.e., the key feature determining succulence, is maximised (Nuernbergk 1961). As mentioned above, cuticular transpiration barrier properties may be of great importance for the optimisation of CAM function, but this feature has only recently gotten attention in the context of CAM (Messerschmid et al. in press). To quantify the water permeability of leaf surfaces, minimum leaf conductance (*g*_min_) can be calculated from simple leaf drying curves (Burghardt and Riederer 2003). Apart from these water‐conserving properties, water‐capturing traits such as shallow root systems, specialised emergences or tanks, transpiration‐reducing traits such as indumentum, leaf shape and growth form and life history may also play a role in shaping the CAM syndrome in a particular plant lineage. Therefore, an integrative approach that includes CAM‐associated traits is needed to understand the evolution of CAM.

Species of the Macaronesian genus *Aichryson* have never been considered ‘typical’ CAM plants, let alone strong CAM plants, despite the genus belonging to the emblematic Crassulaceae, which also contains the CAM model genus *Kalanchoe* (e.g., Hartwell et al. 2016; Yang et al. 2017; Moseley et al. 2018) and numerous other documented CAM plants (Messerschmid et al. 2021). *Aichryson* is monophyletic and closely related to the Macaronesian genera *Monanthes* and *Aeonium* (Messerschmid et al. 2020), both of which contain several strong CAM species (Lösch 1990). Kim et al. (2008) estimated the age of the most recent common ancestor (MRCA) of *Aichryson*, *Monanthes* and *Aeonium* to be 10.23–6.93 Ma; however, Messerschmid et al. (2023) inferred an older age, a time when most of the Canary Islands were present, except for the westernmost islands La Palma and El Hierro (Zaczek et al. 2015). The age of the MRCA of *Aichryson* has been reported to be approximately 4.25 ± 2.69 Ma (Messerschmid et al. 2023), and the genus appears to have colonised all Canary Islands as well as Madeira and the Azores Island Santa Maria within a short time (Kim et al. 2008). The 15 currently recognised species of *Aichryson* cover a wide climatic range, occurring in the desert‐like eastern Canary Islands Fuerteventura and Lanzarote, as well as on the five western Canary Islands, which are wetter and have a greater variety of vegetation types, and Madeira and the Azores (Bañares‐Baudet 2002, 2015b, 2017; Moura et al. 2015). While the western Canary Islands (especially La Palma) host a large number of *Aichryson* species, only three species ( *A. tortuosum* with its two subspecies, subsp. *tortuosum* and subsp. *bethencourtianum*, *A. pachycaulon* subsp. *pachycaulon* and *A. laxum* subsp. *laxum*) are present on the eastern Canary Islands. The genus *Aichryson* is taxonomically divided into two sections: section *Aichryson* and section *Macrobia*. Section *Macrobia* comprises only *A. tortuosum* with its two subspecies, both of which are perennial and often display a characteristic purplish‐red tinge (Eggli 2003). *Aichryson tortuosum* subsp. *tortuosum* is distinguished by its sessile leaves with short glandular hairs excreting a viscid substance, whereas subsp. *bethencourtianum* also has sessile leaves but bears longer, non‐glandular hairs. All remaining species of the genus belong to section *Aichryson* and are monocarpic, typically annual or short‐lived (biennial to rarely triennial) plants (Bañares‐Baudet 2015b). Intensive studies of ten species (Lösch 1990) with gas‐exchange and titratable‐acidity measurements on plants exposed to daytime temperatures of 10°C, 15°C, 20°C, 25°C and 30°C revealed low positive nocturnal CO_2_ assimilation rates, especially at high temperatures, in seven species of *Aichryson* (Table 1). The maximum assimilation rate differed strongly between species (ranging from about 20 to 180 mg CO_2_ d^−1^ g^−1^ dry mass) and the maximum was reached at different, overall relatively cool temperatures, that is, 10°C, 15°C or 20°C. Furthermore, the species differed in CAM‐related and leaf hydraulic traits such as degree of succulence, leaf mass per area (LMA), indumentum and density and distribution of stomata. Lösch (1990) interpreted his findings in an eco‐evolutionary framework in which he identified two fundamentally different ecophysiological strategies, namely (1) a slow‐growing, shrubby, perennial lifestyle with highly succulent leaves and an overall low assimilation rate, which, at temperatures above 25°C, is achieved predominantly by CAM photosynthesis, and (2) a fast‐growing, annual or short‐lived lifestyle with weakly succulent leaves and an overall high assimilation rate over a broad temperature range, which is achieved by diurnal or diurnal plus nocturnal CO_2_ assimilation. A recent phylogenomic study of *Aichryson* revealed that the perennial *A. tortuosum* , representing Lösch's strategy 1, is nested among the short‐lived species (Hühn et al. 2022), indicating a secondary shift towards the perennial lifestyle and stronger CAM expression. In contrast, the most basal lineages of *Aichryson*, such as *A. laxum* subsp. *laxum*, *A. laxum* subsp. *latipetalum* and *A. palmense,* are also short‐lived species, supporting the idea that strategy 2 is ancestral in the genus. The strict annuals *A. parlatorei* and *A. punctatum* are found in derived positions of another clade of *Aichryson* (Hühn et al. 2022), suggesting divergent ecological adaptation. These shifts in ecophysiological strategy and their relation to the broad range of humid to arid environments in its distribution range render *Aichryson* a highly suitable study group to investigate the evolutionary change of CAM and CAM‐related traits with a strong experimental component to reveal further evidence for the CAM continuum in plant lineages with predominant C_3_ photosynthesis and weak CAM.

**TABLE 1:** Currently accepted taxa of *Aichryson* (following Nyffeler 2003, Bañares‐Baudet 2015a, 2015b, 2017, and Moura et al. 2015), their ploidy levels according to Hühn et al. (2022), their distribution in Macaronesia (A = Azores, C = Gran Canaria, F = Fuerteventura, G = La Gomera, H = El Hierro, L = Lanzarote, M = Madeira, P = La Palma, T = Tenerife) and the methods by which they were sampled in this study (Ploidy level, Distribution, Ancestral State Reconstruction, CAM Performance Experiment, δ^13^C Mean/SD values from carbon isotope analysis), indication of CAM according to Lösch (1990). For additional details on each accession, see Tables S1 and S2.

Section	Species	Subspecies	Ploidy	Distribution	Ancestral State Reconstruction	CAM Performance Experiment	δ^13^C	Lösch (1990)
*Aichryson*	*A. bituminosum* Bañares		4x	C	X		−31.60 ± 0.74 (*n* = 2)	
	*A. bollei* Webb ex Bolle		4x	P	X	X	−28.85 ± 3.86 (*n* = 8)	no CAM detected^a^
	*A. brevipetalum* Praeger		4x	P	X		−23.85 ± 3.05 (*n* = 3)	
	*A. divaricatum* (Aiton) Praeger		?	M	X		−27.37 ± 3.50 (*n* = 3)	
	*A. dumosum* (Lowe) Praeger		?	M	X	X		
	*A. laxum* (Haw.) Bramwell	subsp. *latipetalum* Bañares & M.Marrero	2x	T	X		−30.34 ± 0.39 (*n* = 2)	
		subsp. *laxum*		H, P, G, T, C, F	X		−29.91 ± 2.50 (*n* = 25)	no CAM detected^a^
	*A. pachycaulon* Bolle	subsp. *gonzalezhernandezii* (G.Kunkel) Bramwell	4x	G	X		−31.27 ± 3.63 (*n* = 3)	
		subsp. *immaculatum* (Webb ex Christ) Bramwell	4x	T	X		−30.78 ± 1.95 (*n* = 3)	no CAM detected^a^
		subsp. *pachycaulon*	4x	F	X		−26.81 ± 0.85 (*n* = 2)	
		subsp. *parviflorum* (Bolle) Bramwell	4x	P	X		−32.92 ± 2.54 (*n* = 3)	
		subsp. *praetermissum* Bramwell	4x	C	X		−29.21 ± 3.38 (*n* = 5)	
	*A. palmense* Webb ex Bolle		2x	P	X		−29.13 ± 1.80 (*n* = 8)	C_3_‐CAM
	*A. parlatorei* Bolle		4x	H, P, G, T, C	X		−29.69 ± 2.31 (*n* = 11)	C_3_‐CAM
	*A. porphyrogennetos* Bolle		3x(?)	C	X		−26.85 ± 2.74 (*n* = 9)	C_3_‐CAM
	*A. punctatum* (C.Sm. ex Link) Webb & Berthel.		4x	H, P, G, T, C	X		−27.86 ± 3.45 (*n* = 9)	C_3_‐CAM
	*A. roseum* Bañares		4x	C	X	X	−31.72 ± 2.32 (*n* = 5)	
	*A. santamariensis* M. Moura, Carine & M.Seq.		?	A				
	*A. villosum* (Aiton) Webb & Berthel.		?	M	X		−28.54 (*n* = 1)	C_3_‐CAM
*Macrobia*	*A. tortuosum* (Aiton) Webb & Berthel.	subsp. *bethencourtianum* (Bolle) Bañares	2x	F	X	X	−22.99 ± 0.11 (*n* = 2)	C_3_‐CAM
		subsp. *tortuosum*	2x	L	X	X	−24.55 (*n* = 1)	C_3_‐CAM

^a^ Lösch (1990) reported no measurable CAM activity under any tested conditions.

This study builds on three previous studies of *Aichryson*, that is, (1) physiological work by Lösch (1990) with gas‐exchange and titratable‐acidity measurements as well as assessment of stomatal density and succulence for several species, (2) a well‐sampled and resolved phylogeny of the genus by Hühn et al. (2022) and (3) 99 carbon isotope measurements yielding multiple δ^13^C values for nearly all species (Messerschmid et al. 2021). To this, we add data on the distribution, habitat preference and biogeography of *Aichryson*, as well as titratable‐acidity data for more than half of all species. Additionally, we include a comparative climate‐chamber experiment, which includes drought and temperature treatments, for five selected taxa representing different ecological strategies within the genus. These taxa cover contrasting island origins (Madeira vs. the Canary Islands), life forms (short‐lived to perennial) and growth strategies, ranging from the fast‐growing *A. bollei* and the disturbance‐tolerant *A. roseum* to the more succulent, perennial subspecies of *A. tortuosum* . Together, they capture the ecological range of *Aichryson* from mesic to seasonally dry habitats, providing a representative framework to assess photosynthetic responses under controlled environmental conditions. In addition, leaf *g*_min_ was determined for the same five taxa for the first time. To explore how climatic factors may have shaped the diversification and ecological differentiation of *Aichryson* across Macaronesia, we analysed species distributions in relation to environmental space. Given the strong geographic structuring and high rate of island endemism within the genus, we hypothesised that species occupy distinct climatic niches reflecting both conditions on the islands and evolutionary history. We hypothesise that from an ancestral mesic ecology with a predominantly C_3_ physiology and low‐level CAM capacity, several ecophysiological shifts occurred in response to the water availability of the respective habitats. More pronounced CAM activity evolved in the strongly succulent and perennial *A. tortuosum* lineage (including subsp. *bethencourtianum*) distributed on the eastern Canary Islands in adaptation to arid conditions. A reversal to predominant C_3_ photosynthesis occurred in a lineage that dispersed into the wetter climatic conditions of Madeira. We hypothesise that the fast‐growing, annual lifestyle with high diurnal and nocturnal assimilation rates described by Lösch (1990) represents a divergent, derived ecological strategy to cope with seasonal drought.

## 2 Materials and Methods

### 2.1 Taxon Sampling, Occurrence Data and Phylogenetic Framework

Our study includes all 15 currently described species of *Aichryson*. All sampled taxa are listed in Table 1 along with additional information and an island‐level overview of their distribution. Occurrence data for each taxon were compiled from various sources, including herbarium records, a field expedition to the Canary Islands, the Global Biodiversity Information Facility (GBIF) and published literature. A detailed summary of distribution data is provided in Table S1.

Trait data were compiled from multiple sources: ploidy and the phylogeny were obtained from Hühn et al. (2022); already existing gas‐exchange measurements and nocturnal acid accumulation (ΔH^+^) across different temperature regimes from Lösch (1990), and δ^13^C values from Messerschmid et al. (2021) (Table S2). As a phylogenetic framework, we used the most recent species‐level phylogeny of *Aichryson* based on RADseq data from Hühn et al. (2022), which included 29 accessions representing 14 of the 15 currently recognised species. *Aichryson santamariensis*, endemic to the Azores, was not included in that phylogeny but is incorporated into our analyses based on ITS data as well as trait and distribution data from other sources (Moura et al. 2015). We pruned the maximum likelihood tree for the analyses presented here to retain only those taxa for which informative trait and distribution data were available. Taxa were excluded if no relevant data were available or if multiple accessions of the same species existed, in which case only one representative was retained. The resulting pruned tree contained 20 taxa (14 species) (Table 1 and Table S1 for sampling details).

### 2.2 Carbon Isotope Measurements and Ancestral State Reconstruction

Carbon isotope composition (δ^13^C) was determined for a total of 105 *Aichryson* samples. Of these, 67 accessions were collected during fieldwork in 2018, and 32 were derived from herbarium material. All 99 samples were analysed using standard procedures in the laboratory of the Applied and Analytical Palaeontology group at the Institute for Geoscience, JGU Mainz (see Messerschmid et al. (2021) for details). In addition, six previously published δ^13^C values from various *Aichryson* species were included from Tenhunen et al. (1982) (Table S2).

An ancestral state reconstruction (ASR) analysis was conducted in R (R Core Team 2025) using the fastAnc() function from the ‘ape’ package (Paradis and Schliep 2019) and the phytools framework (Revell 2024) based on the pruned phylogenetic tree (see previous paragraph). The analysis was performed using a dataset comprising the species‐level median δ^13^C calculated from all available samples per taxon, which avoids bias from outlier measurements. Prior to ASR, we compared four evolutionary models (Brownian Motion (BM), Ornstein–Uhlenbeck (OU), Early Burst (EB) and Pagel's λ) using AICc model selection implemented in fitContinuous() (geiger package; Harmon et al. 2008). Pagel's λ was identified as the best‐fitting model and subsequently used for the main ASR, while BM results were included as a sensitivity analysis. Phylogenetic signal in δ^13^C was quantified using Pagel's λ, and node estimates were reconstructed under the best‐fitting model. Node uncertainty was assessed using 1000 parametric bootstrap replicates of the ASR. Tree visualisation and mapping of δ^13^C values along branches were performed using contMap() in phytools. For visualisation and to standardise branch lengths, the phylogeny was transformed to an ultrametric form using the force.ultrametric() function (phytools; method = ‘extend’), ensuring equal root‐to‐tip distances without altering topology.

### 2.3 Bioclimatic Variables and Principal Component Analysis

In total, 72 *Aichryson* accessions were collected during fieldwork in March and April 2018 on Gran Canaria, Tenerife, La Palma and La Gomera. To avoid duplication, accessions with identical species and coordinates were filtered out, resulting in 66 unique occurrences used for further analysis, representing all taxa native to these islands (Table S1). To expand the dataset, 181 additional herbarium specimens were examined at the Herbarium of the Sukkulenten‐Sammlung Zürich (ZSS), the Herbario del Jardín Botánico Canario ‘Viera y Clavijo’ in Las Palmas, Gran Canaria (LPA), the Herbarium of the Canarian Institute for Agricultural Research, Tenerife (ORT) and the Herbarium of the University of La Laguna, Tenerife (TFC) (Table S1). As the number of records remained too low for many species to perform robust analyses, occurrence data were complemented by georeferenced records extracted from GBIF (Download: https://doi.org/10.15468/dl.ekq7v9; https://doi.org/10.15468/dl.d3mujn). Given the known issues with data quality and potential misidentifications in public databases such as GBIF (Maldonado et al. 2015; Zizka et al. 2020), we applied automated data cleaning using the R package *CoordinateCleaner* v3.0.1 (Zizka et al. 2019), using default settings. This process removed records with likely erroneous coordinates (e.g., biodiversity institutions, centroids, zeros) as well as duplicate records based on identical species names and coordinates. In addition, we verified the taxonomic reliability of the records by comparing their geographic locations with the species' known native distributions, as listed in Plants of the World Online (POWO 2025; https://powo.science.kew.org/). Occurrences falling outside the expected native ranges were excluded. In total, 1119 *Aichryson* occurrences were compiled (Table S1).

For each occurrence, values for 19 bioclimatic variables (Bio01–Bio19) and elevation were extracted from the WorldClim v2.1 dataset (Fick and Hijmans 2017), which provides historical climate data for the period 1970–2000 at a resolution of ~1 km^2^ (30 arc‐seconds), see Table S3. For 18 records located very close to the coastline, bioclimatic values could not be retrieved because their coordinates fell outside the bounds of the terrestrial raster grid (i.e., into ocean cells). These points were excluded, resulting in a final dataset of 1101 occurrence points used for climatic analyses. All variables were scaled and centred, and a principal component analysis (PCA) was performed using the prcomp() function from the ‘stats’ package in base R (R Core Team 2025) to summarise climatic variation among occurrence points. The first two principal components (PC1 and PC2) were retained for visualisation and further analysis.

To compare the climatic conditions occupied by *Aichryson* species with the climatic space available on each island, a second dataset of randomly distributed background points (max. 2000 points per archipelago) was generated. Tests with the full set of raster cells showed highly similar results, indicating that the climatic envelope was already saturated with 2000 points. Bioclimatic values were extracted for these points and projected into the same PCA space using the loadings and scaling factors from the occurrence‐based PCA. This allowed direct comparison of the realised and available climatic niche space for the Azores, Madeira and Canary Islands. For visualisation, the realised climatic niche of *Aichryson* (based on species occurrences) was plotted as point clouds, and the available climatic space per archipelago was shown using convex hulls around all background points, reflecting the full climatic extent.

To summarise environmental variation among species and identify distinct climatic groups, we conducted a PCA of species‐level mean values for the 19 bioclimatic variables and elevation. Based on the PCA scores, species were grouped using *k*‐means clustering (*k* = 3). To further visualise climatic coherence and variable‐specific patterns within these clusters, we generated a heatmap of *z*‐standardised mean climatic values using Euclidean distances and complete linkage hierarchical clustering implemented in the ‘pheatmap’ package (Kolde and Kolde 2015). In addition, we analysed the full climatic niche structure using all 1101 occurrence records across species. We generated a second heatmap based on occurrence‐level climatic data to visualise environmental overlap among taxa and produced boxplots of each bioclimatic variable per species to show the range and distribution of realised climatic niches beyond species averages (Figures S2 and S3).

To directly compare the environmental conditions associated with different life forms, a two‐dimensional climate space was constructed using raw values of annual mean temperature (Bio01) and annual precipitation (Bio12). This allowed interpretation of the original climatic units and visual separation of short‐lived (≤ 3 years) and perennial taxa. To test for climatic differentiation between life forms, a multivariate analysis of variance (MANOVA) was conducted using the two raw climatic variables.

All spatial processing, calculation of mean climatic values, PCA and cluster analyses were carried out in R v4.4.3 (R Core Team 2025).

### 2.4 Climate‐Chamber Design and Titratable‐Acidity Measurements

The main climate‐chamber experiment focused on five taxa, that is, *Aichryson bollei*, *A. dumosum*, *A. roseum* , *A. tortuosum* subsp. *tortuosum* and *A. tortuosum* subsp. *benthencourtianum*, chosen to capture both major phylogenetic lineages and the range of ecophysiological strategies under strictly controlled environmental conditions. Plants were raised from seeds, except the two *A. tortuosum* subspecies, which were propagated from cuttings of several mother plants. Between 10 and 20 individuals per species were cultivated in 8 × 8 cm pots with standard substrate. The experiment consisted of three consecutive phases: acclimatisation, treatment and reversal. During acclimatisation (4–10 weeks), plants were maintained either in a cool regime (20°C day/10°C night) or a warm regime (30°C/20°C), under a 12‐h photoperiod; irrigation was provided approximately three times per week. At the end of acclimatisation, leaves were sampled at dusk (7:00–8:00 PM) and dawn (6:00–7:00 AM) to quantify baseline ΔH^+^. During the treatment phase (4–5 weeks), two stress regimes were applied: drought, implemented by reducing watering to once per week (yielding cold–dry and warm–dry groups), and temperature switch, implemented by transferring plants from the cool to the warm chamber or vice versa (cold–switch and warm–switch groups). Control plants remained under their initial acclimatisation conditions. At the end of the treatment phase, dusk and dawn sampling were repeated. In the reversal phase (another 4–5 weeks), all plants were returned to their initial acclimatisation conditions and sampled again. Thus, each plant contributed six sampling points (dusk and dawn at the end of each phase).

To broaden taxon coverage while keeping chamber time manageable and without over‐taxing the available plant material, additional species were assessed under a reduced switch–only protocol. For these species, only a few individuals were available and resident in the respective climate chambers: Five taxa, *Aichryson laxum* subsp. *laxum*, *A. pachycaulon* subsp. *gonzalezhernandezii, A. pachycaulon* subsp. *parviflorum, A. porphyrogennetos* and *A. villosum* , in the cool chamber under cold–constant and cold–switch (treatment and reversal) conditions, without dry manipulation. Two taxa, *A. bituminosum* and *A. pachycaulon* subsp. *praetermissum*, analogously in the warm chamber under warm‐constant and warm‐switch (treatment and reversal) conditions, and *A. palmense* only under warm–constant conditions.

From each individual, two to three fully expanded, healthy leaves per plant were collected, immediately weighed, frozen in liquid nitrogen and stored at −20.0°C until analysis. For titration, frozen samples were chopped and extracted in 20% ethanol at 65°C for 60 min. After cooling to room temperature, extracts were aliquoted in three replicates and titrated to neutrality with 0.1 M NaOH using 0.1% bromothymol blue as an indicator.

Based on established thresholds (e.g., Fleck et al. 2026), species were classified as CAM‐inducible using a treatment‐based criterion: we first computed the median ΔH^+^ per treatment (across replicates) and then used the maximum of these treatment medians to represent each taxon. A taxon was deemed inducible if this mean exceeded 10 μmol·g^−1^, or if any single treatment median exceeded 10 μmol·g^−1^ (Table S2).

### 2.5 Minimum Leaf Conductance and Degree of Succulence

To further characterise the five selected taxa included in the main climate‐chamber experiment, their general drought resistance was assessed by measuring minimum leaf conductance (*g*_min_; m·s^−1^) and degree of succulence (g·m^−2^). These traits were selected based on findings from *Aeonium* (Messerschmid et al. in press), which show a clear negative correlation between CAM performance and *g*_min_. Both parameters provide insight into water retention capacity when stomata are closed. For each taxon, six fully expanded leaves were collected from the respective mother plants maintained under controlled greenhouse conditions, independent of the climate‐chamber experiment (Table S2). Drying curves were obtained from detached, rehydrated leaves following Burghardt and Riederer (2003). After sealing the cut bases with molten paraffin wax, leaves were weighed repeatedly to infer transpiration rates, and *g*_min_ was calculated as the mean conductance beyond the turgor‐loss point. The degree of succulence was calculated as the ratio of leaf water mass to hydrated leaf area (Delf 1912). Values were averaged per taxon to obtain representative means.

## 3 Results

### 3.1 Distribution of *Aichryson* (Bioclimatic Variables and PCA)

Most species and subspecies of *Aichryson* are single‐island endemics. For an overview of species distribution and endemism patterns, see Table 1. Only three species are more widespread: *Aichryson laxum* subsp. *laxum* (El Hierro, Fuerteventura, Gran Canaria, La Gomera, La Palma and Tenerife), *A. parlatorei* (El Hierro, Gran Canaria, La Gomera, La Palma and Tenerife) and *A. punctatum* (El Hierro, Gran Canaria, La Gomera, La Palma and Tenerife; Table 1).

**FIGURE 1:**
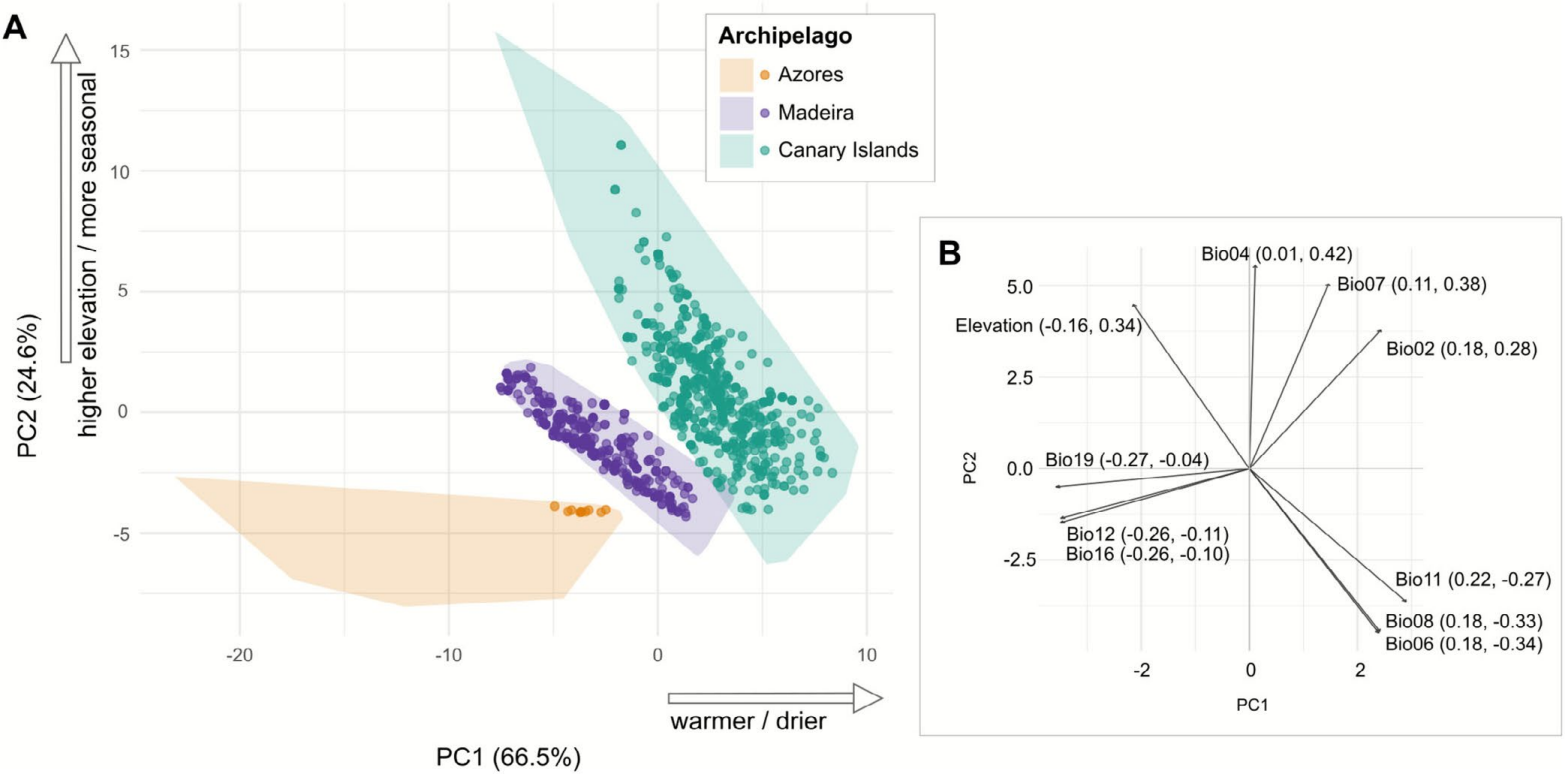
Climatic niche space of *Aichryson* across the Macaronesian archipelagos. (A) Principal component analysis (PCA) based on 19 bioclimatic variables and elevation (WorldClim v2.1) for 1101 georeferenced occurrence records. Shown are the first two principal components, which together explain 91.1% of the total climatic variation (PC1 = 66.5%, PC2 = 24.6%). Each point represents one occurrence record, coloured by archipelago (Azores, Madeira, Canary Islands). Shaded convex hulls delineate the full climatic space available on each archipelago, based on background points. (B) Loadings of the 10 most influential bioclimatic variables (ranked by vector length √(PC1^2^ + PC2^2^)) on the first two axes, with loading values given in parentheses (PC1, PC2). Longer arrows indicate variables that contribute more strongly to climatic differentiation along the PCA axes. Exact loading values for all 19 variables and elevation, including a description of each variable, are provided in Table S3.

Climatic niche variation in *Aichryson* was examined using multivariate analyses based on 19 bioclimatic variables and elevation. A site‐level PCA of all georeferenced occurrence points, coloured by archipelago (Figure 1), revealed distinct environmental spaces for the Azores, Madeira and the Canary Islands. The three archipelagos formed well‐separated clusters in PCA space, with almost no overlap among their respective convex hulls, indicating that the archipelagos differ markedly in their available climatic niches. Records from Madeira were tightly clustered at the lower end of PC2 (24.6% of variance explained) and towards the negative range of PC1 (66.5%), reflecting relatively cool and oceanic conditions. In contrast, occurrence points from the Canary Islands were widely distributed along both axes, except for the quadrant where both PCs are negative, spanning more heterogeneous environmental conditions, including warmer and drier sites as well as higher elevations. Here, elevation refers to the altitude of occurrence records above sea level, and more seasonal corresponds to climatic seasonality in the WorldClim dataset (primarily temperature seasonality, Bio04), with higher values indicating stronger differences between summer and winter conditions. Occurrences from the Azores were restricted to a narrow segment of climatic space, located in the cool, humid portion of the PCA plot (i.e., where both PCs are negative). *Aichryson santamariensis*, the only species present on the Azores, occupies just a small portion of the archipelago's overall climatic space. On Madeira, by contrast, the three endemic species ( *A. divaricatum* , *A. dumosum* and *A. villosum* ) collectively span nearly the full extent of the available niche space (Figure 1, Figure S1). On the Canary Islands, most species are confined to mid‐range positions within the available environmental gradient and do not extend into the more seasonal and highest‐elevation extremes of the Canarian climatic space. A few Canarian occurrence points fall between the Madeira and Canary Islands clusters in PCA space, forming intermediate outliers. These records correspond to species occurring on the northside of La Palma, the northernmost of the western Canary Islands, which may reflect its transitional climatic position relative to Madeira (Figure S1).

**FIGURE 2:**
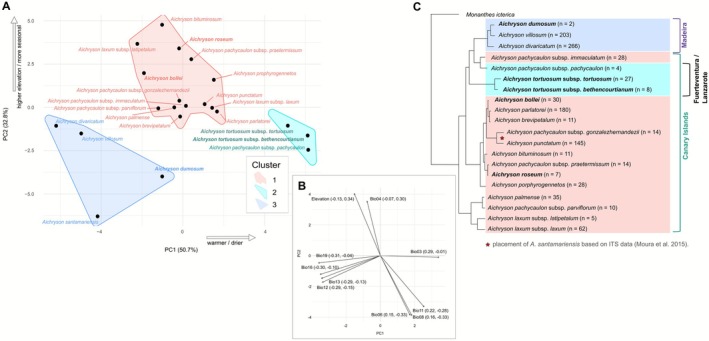
Climatic clustering of *Aichryson* species and its relation to phylogeny. (A) Principal component analysis (PCA) with species‐level mean values of 19 bioclimatic variables and elevation (WorldClim v2.1). The first two principal components explain 83.5% of the total climatic variation (PC1 = 50.7%, PC2 = 32.8%). Each point represents a taxon, with colours indicating *k*‐means clusters (*k* = 3) and convex hulls outlining the boundaries of each climatic cluster. (B) Loadings of the 10 most influential climatic variables on the first two axes, ranked by vector length √(PC1^2^ + PC2^2^). For each variable, loadings on PC1 and PC2 are given in parentheses. Longer arrows indicate stronger contributions to climatic differentiation. Exact loading values for all 19 variables and elevation, including a description of each variable, are provided in Table S3. (C) Pruned maximum likelihood phylogeny of *Aichryson*, based on Hühn et al. (2022), with tip colours corresponding to the PCA clusters shown in (A). Sample sizes per species are indicated in parentheses. Archipelago brackets on the right show the geographic distribution of each species, coloured according to the archipelago clusters defined in Figure 1. *A. santamariensis* was not included in the sampling of Hühn et al. (2022); its phylogenetic placement is indicated based on Moura et al. (2015).

A separate PCA based on species‐level mean values of the 19 bioclimatic variables and elevation (Figure 2A) revealed three clusters of species in the PCA space. PC1 explained 50.7% of the total variance and PC2 32.8%. The clusters were separated along both axes, with each group occupying a distinct region of the plot. Species were grouped into three distinct clusters based on *k*‐means clustering (*k* = 3) and visualised using convex hulls. Cluster 1 includes the majority of species, that is, those native to the western Canary Islands, and is positioned across a broad central range of the PCA space. Cluster 2 consists of three species located towards the warmer and drier end of PC1, all of which occur on Fuerteventura and/or Lanzarote. Cluster 3 comprises four species located in the lower‐left quadrant of the plot, corresponding to negative values for both PCs, and includes taxa restricted to Madeira or the Azores. Given their phylogenetic position, species of clusters 2 and 3 likely originated from ancestors distributed in cluster 1.

**FIGURE 3:**
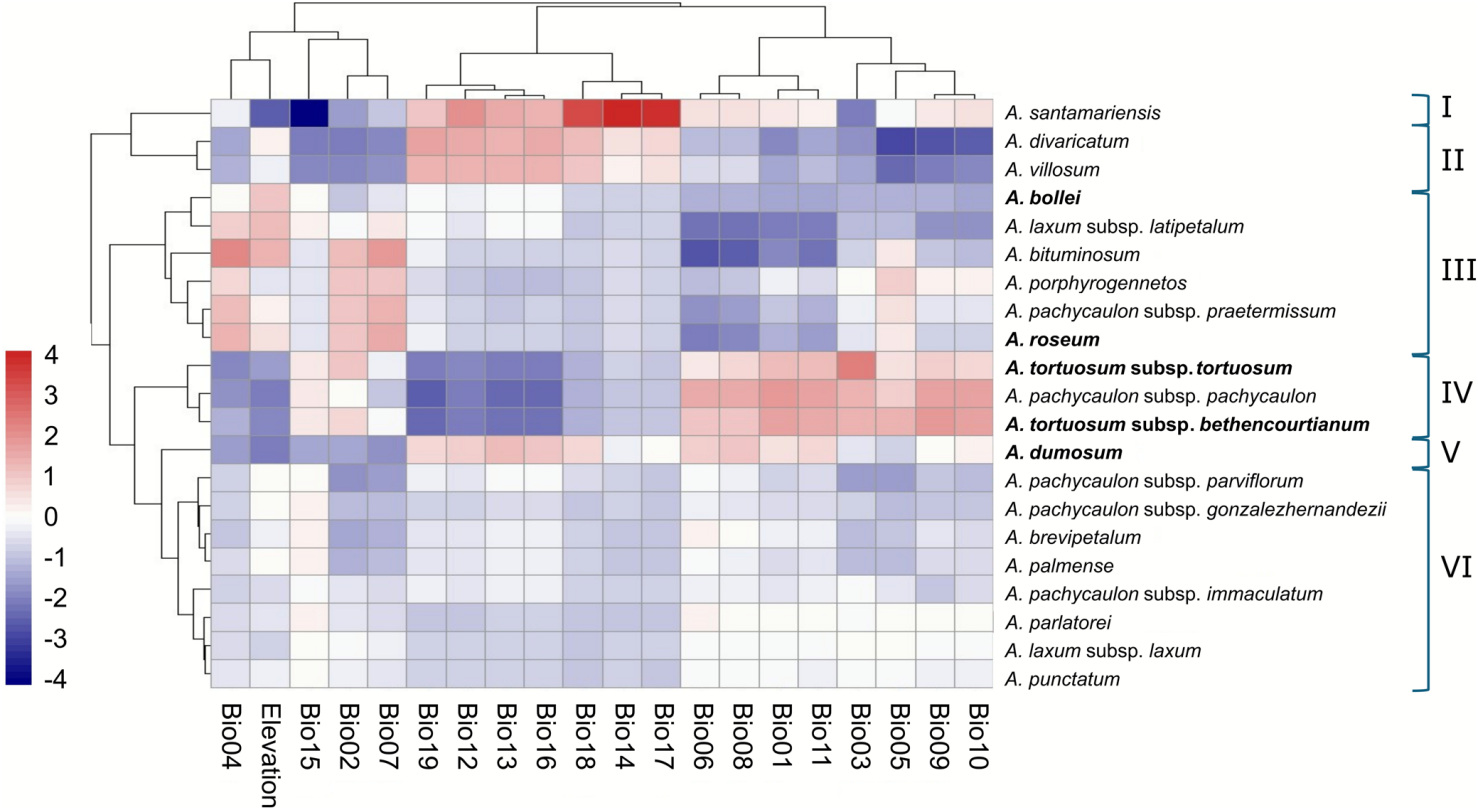
Climatic heatmap of *Aichryson* species. Heatmap based on species‐level means of 19 bioclimatic variables and elevation (WorldClim v2.1), scaled to *Z*‐scores across species. Rows correspond to species and columns to climatic variables, both clustered using hierarchical clustering (Euclidean distance, complete linkage). Colours indicate relative climatic values, with blue representing lower and red higher values compared with the overall mean. Six main species clusters (I–VI) were identified, reflecting distinct climatic niches within the genus. A description of each variable is provided in Table S3.

To complement the multivariate PCA results and further evaluate the climatic consistency of the identified clusters, we additionally visualised species‐level climatic profiles in a heatmap of *z*‐transformed species‐level mean values for the 19 bioclimatic variables and elevation (Figure 3). This approach provides a balanced comparison across species, regardless of sampling density, and highlights the central climatic tendencies of each taxon. The full distribution of occurrences (*n* = 1101) and species‐level variability are shown in Figures S2 and S3. The heatmap revealed distinct clustering of species based on climatic profiles. Among all taxa, *A. santamariensis* showed consistently high *z*‐scores across all precipitation‐related variables and low *z*‐scores for elevation and temperature‐related seasonality measures.

A red‐shaded block in the first group of variables (elevation and seasonality) was observed for multiple species in cluster III (Figure 3). In the PCA based on species‐level mean values (Figure 2), these species are located at the upper part of PC2. This group includes *A. roseum* , *A. porphyrogennetos*, *A. bituminosum* and *A. pachycaulon* subsp. *praetermissum* (all endemic to Gran Canaria), as well as *A. laxum* subsp. *latipetalum* (Tenerife) and *A. bollei* (La Palma). The climatic signals observed in the heatmap are congruent with their positions in PCA space (Figures 1 and 2).

**FIGURE 4:**
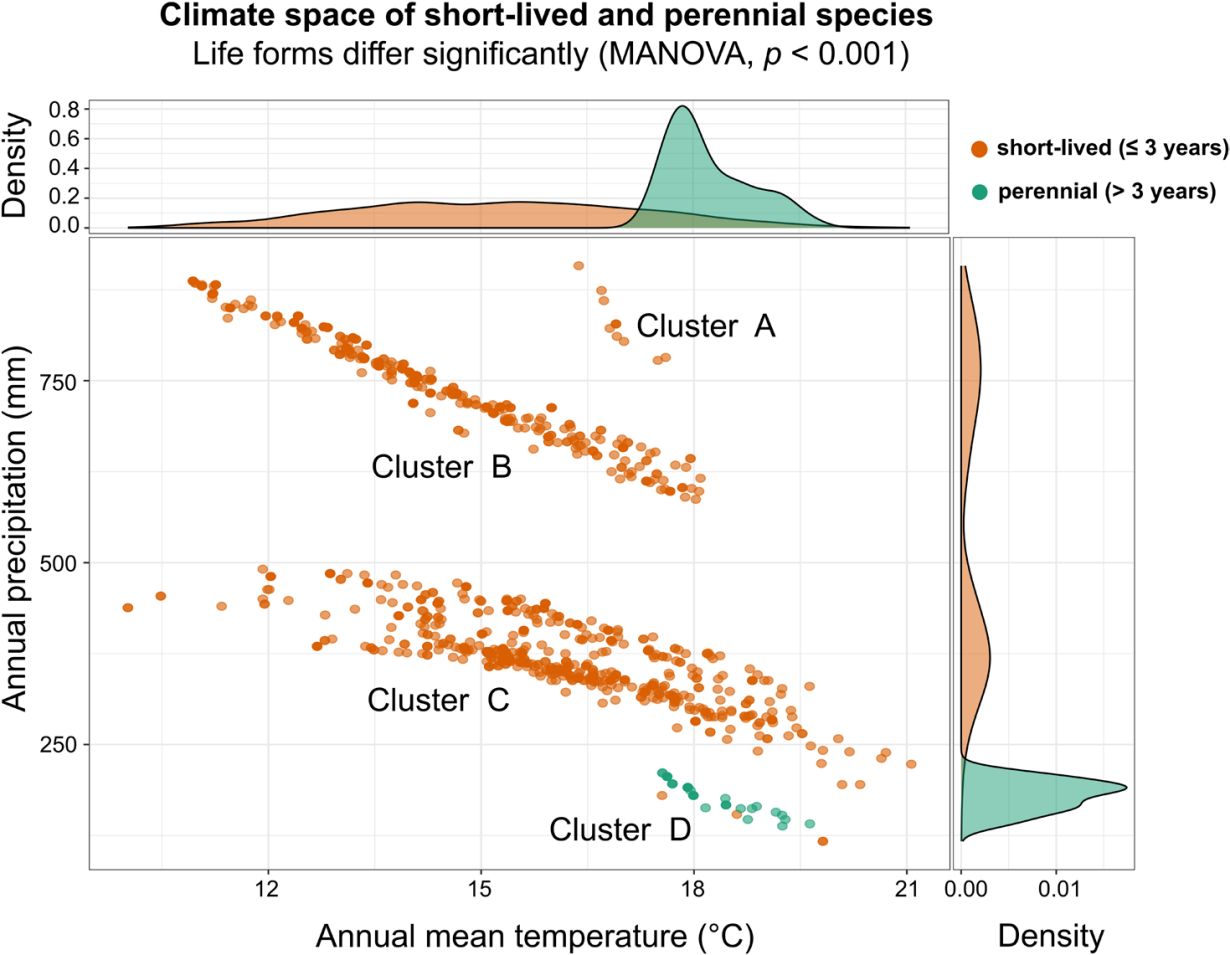
Climatic space occupied by short‐lived taxa (orange) and perennials (green) based on occurrence data. The central scatterplot shows the relationship between annual mean temperature (°C) and annual precipitation (mm), with marginal density plots illustrating the distribution of each life form along the two climatic axes. A multivariate analysis of variance (MANOVA) confirmed a significant difference in climatic niches between life forms (*p* < 0.001). The four clusters visible in the climate space correspond to distinct geographic groupings. Cluster A includes only occurrence points of *A. santamariensis* endemic to the Azores. Cluster B comprises all species endemic to Madeira. Cluster C includes Canary Island species, except those from Fuerteventura and Lanzarote (i.e., Cluster D—the perennial *A. tortuosum* subsp. *tortuosum* and *A. tortuosum* subsp. *bethencourtianum*, and the short‐lived *A. pachycaulon* subsp. *pachycaulon*).

To directly address the environmental conditions associated with different life forms, we compared short‐lived taxa (annuals and short‐lived perennials, ≤ 3 years) with perennial *Aichryson* species in a two‐dimensional climate space based on raw values of annual mean temperature and annual precipitation (Figure 4). Only one species with two subspecies is classified as perennial: *Aichryson tortuosum* subsp. *tortuosum* and *A. tortuosum* subsp. *bethencourtianum*. All remaining species are defined as short‐lived taxa (Bañares‐Baudet 2002, 2015b, 2017; Moura et al. 2015). In contrast to the PCA plots, this representation retains the original climatic units, allowing interpretation of the absolute scale and environmental confines occupied by each group. The comparison of the climatic space between short‐lived and perennial taxa (Figure 4) revealed a clear separation, except for the Fuerteventura short‐lived *A. pachycaulon* subsp. *pachycaulon*. Other short‐lived species were distributed across cooler and wetter environments, while perennial species occupied a narrower range within warmer and drier conditions. Only a group of short‐lived taxa in cluster C fell into the higher temperature range or even exceeded those annual mean temperatures that are also experienced on the eastern Canary Islands (Cluster D). However, a multivariate analysis of variance (MANOVA) confirmed a statistically significant difference in climatic niche occupation between the two life forms (*p* < 0.001). The perennial subspecies of *A. tortuosum* are likely derived from short‐lived ancestors (see Figure 2C).

Together, these results indicate strong climatic structuring within *Aichryson*, with differentiation across life forms, archipelagos and evolutionary lineages.

### 3.2 Evolutionary Reconstruction of Carbon Isotope Ratios

**FIGURE 5:**
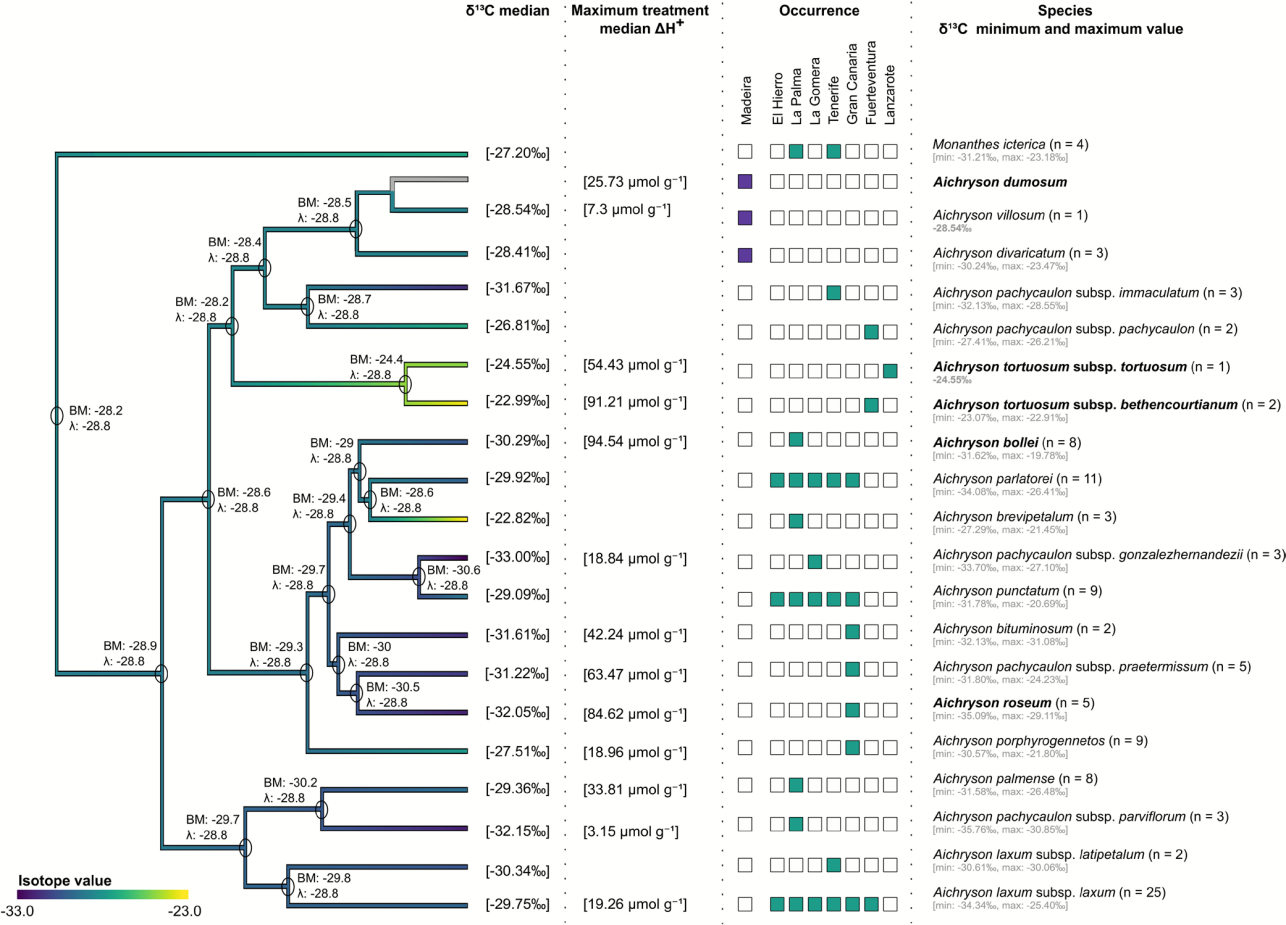
Ancestral state reconstruction (ASR) of the median δ^13^C values across each taxon in *Aichryson*. Tip colours represent observed species‐level median δ^13^C values based on all available replicates. Branch colours indicate reconstructed ancestral δ^13^C values under the best‐fitting evolutionary model (Pagel's λ). ΔH^+^ values (maximum treatment median per species) are shown alongside the phylogeny as quantitative indicators of inducible nocturnal acid accumulation. All individual ΔH^+^ measurements and treatment details are provided in Table S2. Coloured boxes indicate the island occurrence of each taxon. The colours correspond to the archipelago clusters shown in Figure 1. Taxon names are followed by the number of measured samples and their δ^13^C range [minimum, maximum]. The five taxa highlighted in bold were included in a climate‐chamber experiment, allowing direct comparison between δ^13^C values from individuals in the field and physiological responses under controlled conditions.

The δ^13^C dataset comprising the median value for each taxon did not exhibit a significant phylogenetic signal, as indicated by Pagel's λ (Figure 5). Nevertheless, an ASR was performed using the BM model, which enabled visualisation of potential evolutionary trends across the pruned phylogeny.

In total, δ^13^C values for 105 *Aichryson* samples were measured. Median δ^13^C values ranged from −22.82‱ (*A. brevipetalum*) to −33.00‱ (*A. pachycaulon* subsp. *gonzalezhernandezii*). Most values fell within the range typically associated with long‐term C_3_ photosynthesis, as defined by Winter et al. (2005) (Table S2). Only a small number of samples approached δ^13^C values close to −20‱, which are typically associated with a mixture of nocturnal and daytime CO_2_ assimilation. These less negative values were primarily observed in samples collected from drier and more exposed locations, whereas samples from more humid environments showed lower δ^13^C values (Table S2). To evaluate this pattern, we tested for correlations between δ^13^C values and all 19 bioclimatic variables. We used Spearman's rank correlation coefficient (*ρ*) to assess monotonic relationships between variables, independent of linearity. Although none of the variables showed statistically significant correlations (all *p* > 0.05), some weak trends were evident: for example, precipitation of the wettest month (Bio13: *ρ* = −0.16, *p* = 0.124), maximum temperature of the warmest month (Bio05: *ρ* = +0.15, *p* = 0.149) and annual precipitation (Bio12: *ρ* = −0.15, *p* = 0.158). These trends are consistent with the interpretation that δ^13^C values tend to be less negative in warmer and drier environments, potentially reflecting enhanced CAM expression. However, it should be noted that δ^13^C enrichment can also occur in C_3_ plants under water stress due to reduced stomatal conductance and consequently lower discrimination by Rubisco. Figure 5 illustrates the variability of the median δ^13^C values across the genus. The lowest median isotope values were recorded in *A. pachycaulon* subsp. *gonzalezhernandezii*, *A. pachycaulon* subsp. *parviflorum* and *A. roseum* . Conversely, *A. brevipetalum* and both subspecies of *A. tortuosum* exhibited the most positive median δ^13^C values among all species, whereas their respective reconstructed ancestor showed a lower value in the ASR.

### 3.3 Titratable Acidity: Maximum Nocturnal Acid Accumulation Per Taxon

To evaluate CAM activity across species, we used the maximum treatment median of nocturnal acid accumulation (ΔH^+^; dawn minus dusk) per species, representing each taxon's highest observed CAM potential (Figure 5). Taxa showing a ΔH^+^ > 10 μmol·g^−1^ were classified as CAM‐inducible. Titration results revealed considerable interspecific variation in ΔH^+^, with 11 taxa surpassing this threshold (Figure 5). In contrast, *A. villosum* (7.3 μmol·g^−1^) and *A. pachycaulon* subsp. *parviflorum* (3.15 μmol·g^−1^) showed lower values, suggesting very low or no CAM activity. For several taxa, titration data were not available; nonetheless, the results indicate that at least low‐level CAM expression is widespread within *Aichryson*.

### 3.4 CAM Performance Experiment: Nocturnal Acid Accumulation, Minimum Leaf Conductance and Succulence

**FIGURE 6:**
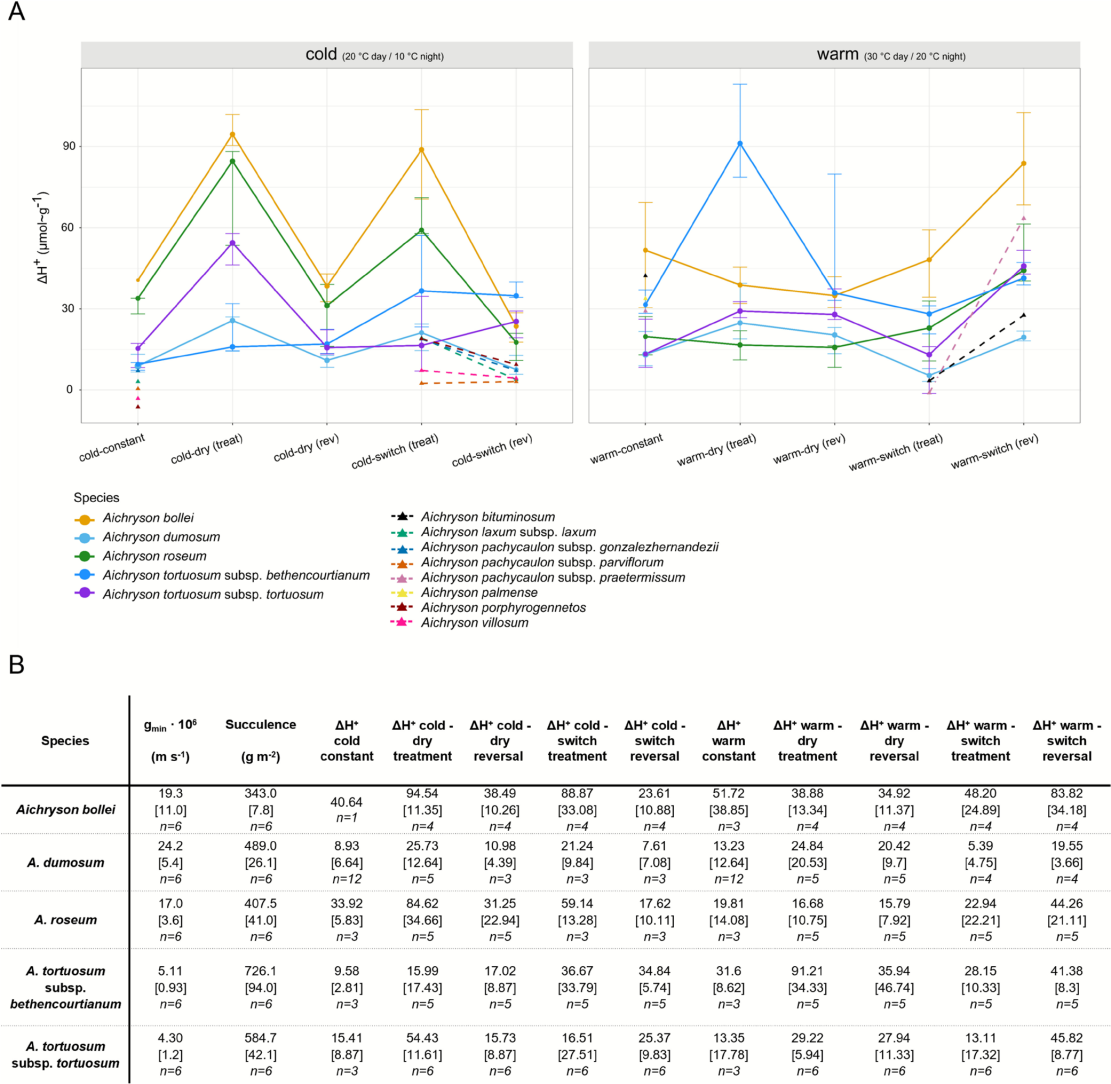
Induction of nocturnal acid accumulation (ΔH^+^) and associated physiological traits in *Aichryson* species under different drought–temperature conditions. (A) Median ΔH^+^ values and interquartile ranges (IQR) for selected species under cold (20°C daytime/10°C night‐time) and warm (30°C daytime/20°C night‐time) conditions across different treatments. Solid lines and circular markers represent the five taxa included in the main climate‐chamber experiment, while dashed lines and triangular markers indicate additional species tested in smaller‐scale experiments. For corresponding data, see Table S2. (B) Summary of physiological parameters for the five taxa included in the main climate‐chamber experiment, including minimum leaf conductance (*g*_min_), succulence, and ΔH^+^ values for each treatment. Values are shown as medians (IQR in brackets) with sample sizes (*n*).

The climate‐chamber experiment revealed notable interspecific variation in physiological traits across treatments and experimental stages (Figure 6). *Aichryson bollei* exhibited the highest values of ΔH^+^, with 40.64 μmol·g^−1^ under constant cold conditions and 51.72 μmol·g^−1^ under constant warm conditions. Under the cold–dry treatment, ΔH^+^ increased to 94.54 μmol·g^−1^ and decreased to 38.49 μmol·g^−1^ after rewatering (cold–dry reversal). Under the cold–switch treatment, ΔH^+^ increased to 88.87 μmol·g^−1^ and dropped again to 23.61 μmol·g^−1^ when plants were returned to cold conditions (cold–switch reversal).

By contrast, *A. dumosum* showed consistently low ΔH^+^ values across all treatments (maximum ΔH^+^ = 25.73 μmol·g^−1^, cold–dry treatment; Figure 6). *Aichryson roseum* showed intermediate ΔH^+^ levels under constant cold conditions (33.92 μmol·g^−1^) but enhanced values under the cold–dry (84.62 μmol·g^−1^) and cold–switch (59.14 μmol·g^−1^) treatments and the warm–switch reversal (44.26 μmol·g^−1^). The low ΔH^+^ levels for some treatments (e.g., warm–dry) for *A. roseum* might point at CAM idling.

The two subspecies of *A. tortuosum* responded differently to the treatments that triggered nocturnal acid accumulation. In *A. tortuosum* subsp. *bethencourtianum*, the warm–dry treatment caused a significant increase in ΔH^+^ from 31.6 μmol·g^−1^ (warm–constant) to 91.2 μmol·g^−1^. In contrast, *A. tortuosum* subsp. *tortuosum* responded most strongly to cold–dry conditions, with ΔH^+^ rising from 15.4 μmol·g^−1^ (cold–constant) to 54.4 μmol·g^−1^, and also showed a marked response during the warm–switch reversal, increasing from 13.1 μmol·g^−1^ to 45.8 μmol·g^−1^ after returning from the cold into the warm chamber.

The minimum leaf conductance (*g*_min_) varied substantially among the tested species, with the short‐lived species ( *A. bollei* , *A. dumosum* and *A. roseum* ) showing a 4‐ to 5‐fold higher *g*_min_ than the perennial taxa (both subspecies of *A. tortuosum* ; Figure 6B). Succulence followed a similar pattern, with the highest value observed in *A. tortuosum* subsp. *bethencourtianum* (726.1 g·m^−2^) and the lowest in *A. bollei* (343.0 g·m^−2^). The perennial species exhibited a distinctly higher succulence than the three short‐lived species.

## 4 Discussion

CAM photosynthesis is a complex and plastic trait with a wide spectrum from weak inducible to strong obligatory forms (Winter 2019). What defines all CAM plants and serves as evidence for CAM activity is the significant nocturnal acidification detected by comparative acid titration of dusk and dawn cell sap (Winter and Smith 2022). A comparison between Lösch (1990) and the present study indicates that only a few *Aichryson* taxa show no measurable CAM activity. In Lösch's work, *A. bollei* , *A. pachycaulon* subsp. *immaculatum* (referred to as *A. pachycaulon* from Tenerife) and *A. laxum* did not express CAM under any tested conditions. In the present study, CAM activity could not be detected in *Aichryson pachycaulon* subsp. *parviflorum* (not sampled by Lösch 1990) and *A. villosum* (very weak facultative CAM in Lösch 1990), as indicated by both titratable acidity and δ^13^C isotope values (Figures 5 and 6). However, only limited plant material was available for these two species, restricting replication. In contrast to Lösch (1990), we found a limited or conditional CAM‐like behaviour in *A. bollei* and *A. laxum* subsp. *laxum*, as indicated by their titratable‐acidity measurements. For *A. pachycaulon* subsp. *immaculatum*, only δ^13^C values could be determined, as the available material was again very limited. The species with predominant C_3_ carbon fixation are scattered across the phylogenetic tree but distributed in distinct climatic clusters, as revealed by the environmental PCA (Figure 2). *Aichryson laxum* subsp. *laxum* and *A. pachycaulon* subsp. *parviflorum* fall into cluster 1 and are located near the centre of the PCA space, indicating intermediate climatic conditions along both temperature and seasonality gradients. This aligns with the habitat and habit of *A. pachycaulon* subsp. *parviflorum* as described by Bañares‐Baudet (2015b), that is, thermo‐mediterranean environments with subhumid to humid ombrotypes and a biennial life form, respectively, suggesting it occupies relatively mild, seasonally moist habitats where CAM may offer limited advantage. *Aichryson villosum*, which showed no CAM expression in the present study, falls within cluster 3, associated with more humid, lower‐elevation environments in Madeira (Figure 2). We therefore assume that the predominantly C_3_ species are evolutionarily derived from C_3_‐CAM ancestors, but now inhabit environments where the ecological advantages of CAM are diminished; likely due to the higher metabolic costs associated with CAM relative to C_3_ photosynthesis (Winter and Holtum 2014).

Although most *Aichryson* species are capable of performing at least some degree of CAM photosynthesis, we discovered clear differences in CAM inducibility and strength, which might be related to different ecophysiological and life history traits of the species. Lösch (1990) hypothesised that *Aichryson* exhibits two distinct ecophysiological strategies. The first is a slow‐growing, shrubby, perennial form characterised by highly succulent leaves and a low overall assimilation rate, primarily relying on CAM photosynthesis at temperatures above 25°C. The second is a fast‐growing, short‐lived form with weakly succulent leaves and a high assimilation rate across a broad temperature range, achieved through diurnal (i.e., the predominantly C_3_ species) or combined diurnal and nocturnal CO_2_ assimilation. In part, our findings can assign the *Aichryson* species to these two main groups, with the two subspecies of *A. tortuosum* belonging to the former and the remaining species to the latter group. However, our results suggest further fine scale differences within both groups.

### 4.1 Isotopic Evidence for CAM Expression and Plasticity

The δ^13^C values per se suggest that most *Aichryson* species rely predominantly on C₃ photosynthesis but may be able to switch between C_3_ and CAM, and this was also inferred as the ancestral physiological state by our ASR analysis (δ^13^C around −28‱, Figure 5). Several taxa, particularly *A. tortuosum* and *A. brevipetalum*, show δ^13^C values close to −20‱ consistent with limited or conditional CAM‐like behaviour (Figure 5, Table S2). Such relatively high δ^13^C values for individual samples may indicate substantial nocturnal carbon assimilation under specific environmental conditions. Another aspect to be considered with δ^13^C measurements is the age and organ of the specimen that is sampled. Seen that most of the short‐lived species grow and flower during the more humid winter and early spring, but extend their seed maturation into the hotter and drier summer (Bañares‐Baudet 2015b), facultative CAM could serve the purpose of completing the seed maturation and thus the plant life cycle, and δ^13^C measurements will consequently depend on the age of the sampled plant material. In fact, Winter and Holtum (2024) have recently emphasised that many of the facultative CAM plants that we know today are annuals and could operate in the CAM mode towards the end of their lives for exactly this reason (see also Winter and Ziegler 1992).

The tendency of less negative δ^13^C values in samples from drier, more exposed sites supports the hypothesis that CAM expression in *Aichryson* may be environmentally induced and represents an adaptive response to water limitation. The δ^13^C values alone are not conclusive, at best indicative. To evaluate whether such physiological tendencies are reflected in species' environmental distributions, we next assessed the climatic niches of *Aichryson* taxa based on occurrence data and bioclimatic variables. This ecological perspective was complemented by our CAM performance experiment (see Chapter 4.3), which aims to experimentally test CAM induction and performance under controlled climate‐chamber conditions.

### 4.2 Climatic Niche Differentiation and Species Groupings

The multivariate analyses revealed distinct patterns of climatic niches among the tested *Aichryson* species, both at the individual and species levels. The PCA of all occurrence points grouped by the archipelago they belonged to (Figure 1) showed that occurrences from Madeira and the Azores were confined to the cooler and wetter part of the climatic space (low PC1 values) and to lower elevations (low PC2 values). This position is consistent with the relatively stable, oceanic climate of Madeira and the Azores, characterised by lower seasonal variation and moderate temperatures (Florencio et al. 2021). In contrast, the broad distribution of Canary Island occurrences mainly across PC2 reflects this archipelago's higher environmental heterogeneity, which is in part caused by a strong elevational gradient and includes more arid lowlands and more pronounced climatic seasonality.

Beyond their differences in photosynthetic behaviour, the *Aichryson* species also vary considerably in their climatic niche breadth (Figure S1). *Aichryson laxum*, for example, occurring on several islands, occupied a wide area in PCA space, suggesting a broad ecological amplitude and tolerance of varying climatic conditions (Figure S1). Conversely, species like *A. villosum* and *A. bollei* exhibit narrower climatic niche spaces, potentially indicating more specialised environmental requirements and being restricted to single islands.

The species‐level PCA and subsequent clustering analysis identified three distinct climatic niche groups (Figure 2A). The first cluster included most species and is associated with a wider range of climates on the western Canary Islands, while the second cluster ( *A. tortuosum* and *A. pachycaulon* subsp. *pachycaulon*) comprised species from hotter and drier conditions on the eastern Canary Islands. The third cluster ( *A. divaricatum* , *A. dumosum*, *A. santamariensis* and *A. villosum* ) was composed of species occupying cooler, low‐elevation environments on Madeira and the Azores. The outgroup, *Monanthes icterica* (Mort et al. 2002), is distributed on the western Canary Islands (Bañares‐Baudet 2015b). Therefore, species of cluster 1 likely represent the ancestral conditions from which lineages independently colonised two climatically divergent regions: one wetter and cooler region, represented by two independent colonisations of the northern Macaronesian islands (Madeira and the Azores), and one drier and warmer region, through two separate colonisations of the eastern Canary Islands. In Madeira, *A. dumosum*, *A. villosum* and *A. divaricatum* represent an early radiation (Figure 2C), while the Azorean *A. santamariensis* originated independently and was not derived from the Madeiran lineage (Moura et al. 2015). Similarly, in the eastern Canary Islands, *A. pachycaulon* subsp. *pachycaulon* and the two subspecies of *A. tortuosum* (i.e., subsp. *tortuosum* and subsp. *bethencourtianum*) likely represent two separate colonisation events from the ancestral pool within cluster 1.

We found a significant climatic separation between short‐lived taxa and perennials in *Aichryson* (Figure 4), except for *A. pachycaulon* subsp. *pachycaulon*, which clustered together with the perennial taxa. This suggests that life history strategies are closely linked to environmental conditions in the genus. Short‐lived taxa appear to be associated with cooler and more mesic habitats, whereas perennials (i.e., both subspecies of *A. tortuosum* ) are more common in warmer and drier areas. This pattern may also reflect the adaptive differentiation in water‐use strategies, phenology, or physiological traits and further supports the idea of strong climatic structuring across the genus, with the perennial life form being the derived condition.

During glacial maxima of the Quaternary, the eastern Islands likely provided a climate more similar to the western Islands, with suitable conditions for *Aichryson* (Ortiz et al. 2006). We hypothesise that this eastern Island lineage of the genus arose while the genus colonised larger areas on the eastern Islands during this time. While most populations went extinct during the last interglacials, some might have adapted to the warmer and drier conditions.

Similar to the recent study of the closely related *Aeonium* (Messerschmid et al. in press), we tested in our CAM performance experiment (see next chapter) whether the colonisation of these significantly wetter (Madeira and the Azores) or drier regions (eastern Canary Islands) and/or the shift in life form is reflected in the ecophysiology of the respective species.

### 4.3 CAM Performance Experiment: Physiological Strategies Under Drought and Temperature Stress

The variation in ΔH^+^ across the different experimental treatments indicates varying degrees of CAM plasticity among the five tested *Aichryson* taxa, reflecting underlying differences in ecophysiological strategy. As originally proposed by Lösch (1990), *Aichryson* species can be broadly grouped into two physiological types: (1) both subspecies of *A. tortuosum* represent the perennial, more CAM‐reliant type with low growth rates; (2) all other *Aichryson* species exhibit higher growth rates and show a C_3_ physiology with low‐level CAM capacity. Coming back to the former physiological type, CAM expression is not uniform in the two subspecies of *A. tortuosum* , suggesting variability of CAM expression within this type. Particularly, the low ΔH^+^ value observed in *A. tortuosum* subsp. *tortuosum* under the warm–switch treatment may reflect higher metabolic flexibility or stress sensitivity. While *A. tortuosum* subsp. *bethencourtianum* generally maintained moderate ΔH^+^ values across treatments, it showed a pronounced increase under combined heat and drought stress (91.21 μmol·g^−1^). *Aichryson tortuosum* subsp. *tortuosum* showed a stronger increase under cold–dry conditions (54.43 μmol·g^−1^) and the warm–switch reversal (45.82 μmol·g^−1^). Such differences and flexibility imply intraspecific divergent responsiveness to stress, which might be caused by local adaptation. It also shows that even species with a high capacity of CAM expression may downregulate CAM and operate in the less costly C_3_ mode when environmental stress is relaxed. Even the most succulent and xeromorphic species of the entire Macaronesian radiation of Crassulaceae, *Aeonium nobile*, may exhibit inconspicuous ΔH^+^ values under relaxed environmental conditions (Messerschmid et al. in press).

*Aichryson bollei* exhibited relatively high ΔH^+^ values across all treatments, indicating a potential for CAM induction under stress conditions. However, the δ^13^C values range mostly between −28‱ and −32‱, typical for C_3_ plants, with only a single outlier (−19.78‱) suggesting enhanced nocturnal carbon fixation under unknown environmental circumstances. In combination with a relatively high *g*_min_, this indicates that *A. bollei* does not exhibit traits of an obligate CAM plant but rather shows limited or conditional CAM‐like behaviour under drought or temperature stress. Being endemic to La Palma, *A. bollei* typically inhabits relatively dry pine forests but can occasionally occur in more humid Monteverde habitats (Bañares‐Baudet 2015b). Its short life cycle of 1–2 years may confer an adaptive advantage in such variable environments by enabling rapid completion of its growth cycle under fluctuating moisture conditions. The discrepancy with Lösch (1990), who found no CAM activity in *A. bollei* , may relate to the taxonomic uncertainty that existed prior to the clarification of this species by Bañares‐Baudet (2002).

In contrast, *A. dumosum* exhibited little variation in ΔH^+^ values across all treatments, none of them exceeding 30 μmol·g^−1^. It is a strictly short‐lived species (Lösch 1990) and therefore may have more time for the completion of its life cycle compared to the faster‐growing *A. bollei* (Nyffeler 2003). It is also a species of the more humid and less seasonal Madeiran realm (Figure 1). These considerations suggest that a more mesic climate and relaxed demands on growth rates may have reduced the overall CAM activity in this species.

*Aichryson roseum* showed intermediate CAM expression, demonstrating clear increases under stress treatments (except for the drought treatment under high temperatures) and indicating some degree of physiological plasticity. It is ecologically and morphologically similar to the above‐discussed *A. bollei* , but is an endemic of Gran Canaria instead of La Palma. It inhabits the potential zones of the Monteverde forests but is usually found in more disturbed habitats that have been turned into open or shrubland vegetation by human activity. Gran Canaria is the most deforested of the Canary Islands (Morales et al. 2009), and there is a possibility that *A. roseum* now does not predominantly occur in those habitats that it originally evolved into. We might therefore see lower CAM activity in this species due to a past adaptation to more humid climates that is not reflected by its current distribution anymore.

Additional traits such as *g*_min_ and succulence may also be distinct for different species. The two perennial taxa were more succulent than the annuals, and this difference was most pronounced between the perennial *A. tortuosum* subsp. *bethencourtianum* (726.1 g·m^−2^) and the annuals *A. bollei* and *A. roseum* (343 g·m^−2^ and 407.5 g·m^−2^, respectively). At the same time, the perennials exhibited much lower *g*_min_ values, which is indicative of more effective cuticular transpiration barrier properties and potentially increased stomatal control and may result in optimised water‐use efficiency under stress. This pattern supports the view that CAM plasticity in *Aichryson* is functionally integrated with leaf‐level water‐saving traits, as was proposed for *Aeonium* (Messerschmid et al. in press), and varies both between species and between life history strategies. In a recent study of *Aeonium*, Messerschmid et al. (in press) show that *g*_min_ was significantly lower, and thus the cuticular transpiration barrier was more effective, in the leaves of the perennial species prevailing in *Aeonium* in comparison to the biennial *Aeonium glandulosum* and *A. tabuliforme*. This is in line with a meta‐analysis of all *g*_min_ values that had been published up until 2017 (Schuster et al. 2017), which further discusses the ecological significance of transpiration barrier properties for the longevity of leaves.

## 5 Conclusion and Future Research

The genus *Aichryson* shows clear climatic structuring across its range, with both the breadth and position of the occupied niche varying notably among species and archipelagos. The range expansion of the genus proceeded from the western Canary Islands twice towards both more humid regions (Madeira and the Azores) and also twice towards more arid environments in the eastern Canary Islands. Concerning CAM evolution in the climatically divergent lineages we observed two evolutionary trajectories: Dispersal to regions with high precipitation and low temperature seasonality led to reduction or loss of CAM inducibility while dispersal to regions with higher aridity and seasonality led to the evolution of stronger CAM capacity complemented by a syndrome of stronger succulence, lower *g*_min_ and perennial lifestyle. Together, these findings highlight a complex interplay between climatic adaptation, physiological evolution and insular biogeography. They provide a basis for further exploration of ecological differentiation, physiological plasticity and evolutionary responses to environmental change in island plant lineages under changing environmental conditions.

## Author Contributions

**Jessica A. Berasategui:** conceptualization (lead), data curation (lead), formal analysis (lead), funding acquisition (supporting), investigation (equal), methodology (lead), project administration (equal), visualization (lead), writing – original draft (lead), writing – review and editing (lead). **Thibaud F. E. Messerschmid:** formal analysis (supporting), investigation (supporting), methodology (supporting), writing – review and editing (supporting). **Stefan Abrahamczyk:** investigation (supporting), writing – review and editing (supporting). **Ángel Bañares‐Baudet:** data curation (supporting), investigation (supporting), supervision (supporting), writing – review and editing (supporting). **Nadine Bobon:** investigation (supporting), writing – review and editing (supporting). **Gudrun Kadereit:** conceptualization (lead), funding acquisition (lead), investigation (equal), project administration (equal), resources (lead), supervision (lead), writing – original draft (lead), writing – review and editing (supporting).

## Conflicts of Interest

The authors declare no conflicts of interest.

## Supporting information


**Figure S1:** Principal component analysis (PCA) of the climatic niche space of *Aichryson* occurrence records, based on 19 bioclimatic variables and elevation. Each point represents a locality, coloured by species. The first two principal components explain 66.5% (PC1) and 24.6% (PC2) of the total variance. PC1 primarily reflects a gradient from cooler, wetter climates to warmer, drier conditions, while PC2 separates localities along a gradient of increasing elevation and climatic seasonality. The plot illustrates clear climatic differentiation among species across their native ranges.
**Figure S2:** Climatic heatmap of all *Aichryson* occurrence records. Heatmap based on raw climatic values (19 bioclimatic variables and elevation; WorldClim v2.1) extracted for 1101 cleaned occurrence points across all species. Variable descriptions are provided in Table S3.
**Figure S3:** Boxplots for each bioclimatic variable per species. Variable descriptions are provided in Table S3.

**Table S1:** (i) Accession Details, (ii) Occurrence Herbaria + Fieldwork, (iii) Occurrence GBIF.

**Table S2:** (i) Carbon isotope measurements, (ii) climate‐chamber experiments, (iii) median acidity, (iv) CAM inducibility summary, (v) *g*_min_.

**Table S3:** (i) Description of the environmental variables used in this study, (ii) PCA loadings for all 19 bioclimatic variables and elevation describing the climatic niche space of *Aichryson* across the Macaronesian archipelagos (Figure 1), (iii) PCA loadings for all 19 bioclimatic variables and elevation based on species‐level means (Figure 2).

## Data Availability

The data that support the findings of this study are available in the Supporting Information of this article.
