# Supplementary material for: Evolutionary Changes in Crassulacean Acid Metabolism (CAM) and Related Traits During the Diversification of Aichryson (Crassulaceae) on the Macaronesian Islands
Source: Ecol Evol. 2026 Jan 2;16(1):e72864. doi: 10.1002/ece3.72864 (PMC12758979; doi:10.1002/ece3.72864)
Supplement: Supplementary file 1 — Figure S1: Principal component analysis (PCA) of the climatic niche space of Aichryson occurrence records, based on 19 bioclimatic variables and elevation. Each point represents a locality, coloured by species. The first two principal components explain 66.5% (PC1) and 24.6% (PC2) of the total variance. PC1 primarily reflects a gradient from cooler, wetter climates to warmer, drier conditions, while PC2 separates localities along a gradient of increasing elevation and climatic seasonality. The plot illustrates clear climatic differentiation among species across their native ranges. Figure S2: Climatic heatmap of all Aichryson occurrence records. Heatmap based on raw climatic values (19 bioclimatic variables and elevation; WorldClim v2.1) extracted for 1101 cleaned occurrence points across all species. Variable descriptions are provided in Table S3. Figure S3: Boxplots for each bioclimatic variable per species. Variable descriptions are provided in Table S3. [file ECE3-16-e72864-s001.pdf]

Supplementary Figure S1

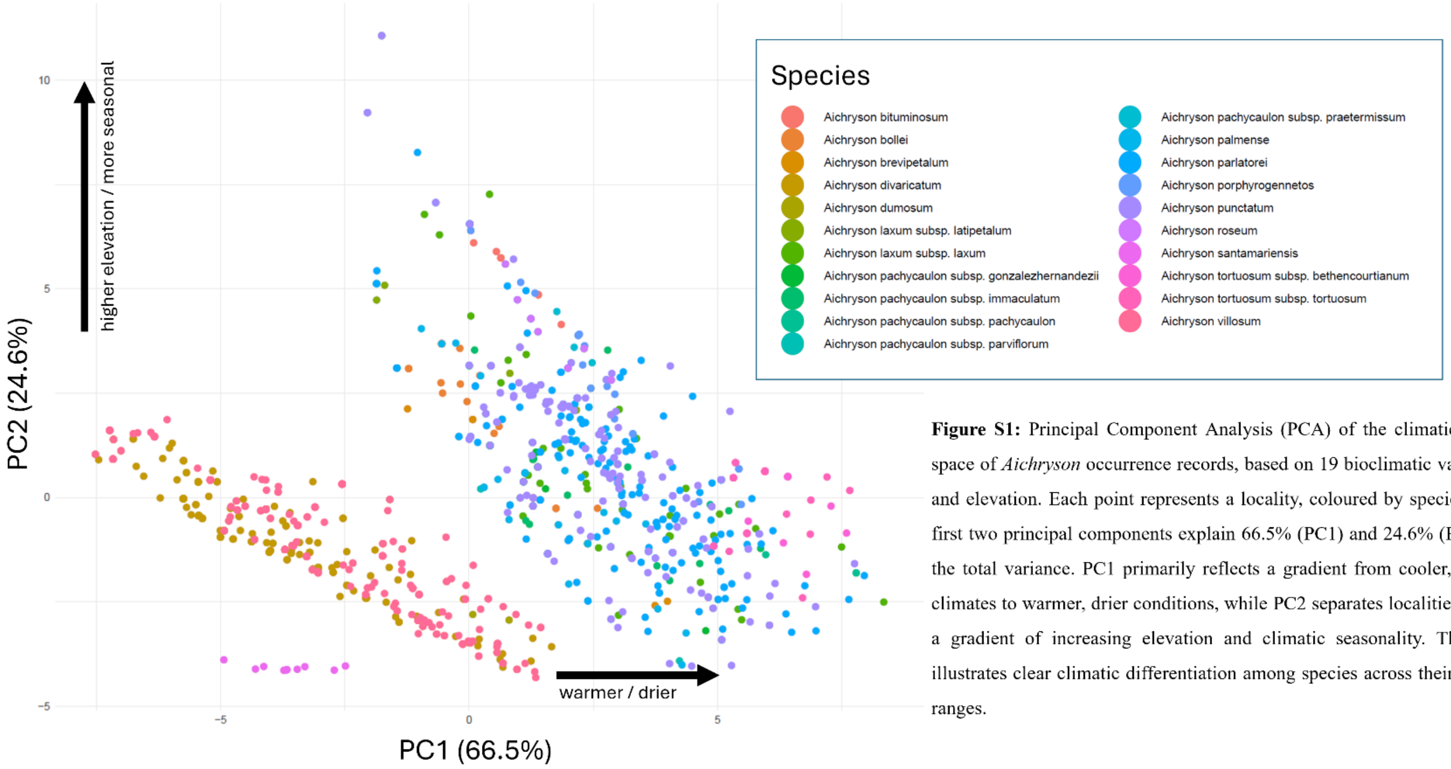

Figure S1: Principal Component Analysis (PCA) of the climatic niche space of *Aichryson* occurrence records, based on 19 bioclimatic variables and elevation. Each point represents a locality, coloured by species. The first two principal components explain 66.5% (PC1) and 24.6% (PC2) of the total variance. PC1 primarily reflects a gradient from cooler, wetter climates to warmer, drier conditions, while PC2 separates localities along a gradient of increasing elevation and climatic seasonality. The plot illustrates clear climatic differentiation among species across their native ranges.

Supplementary Figure S2

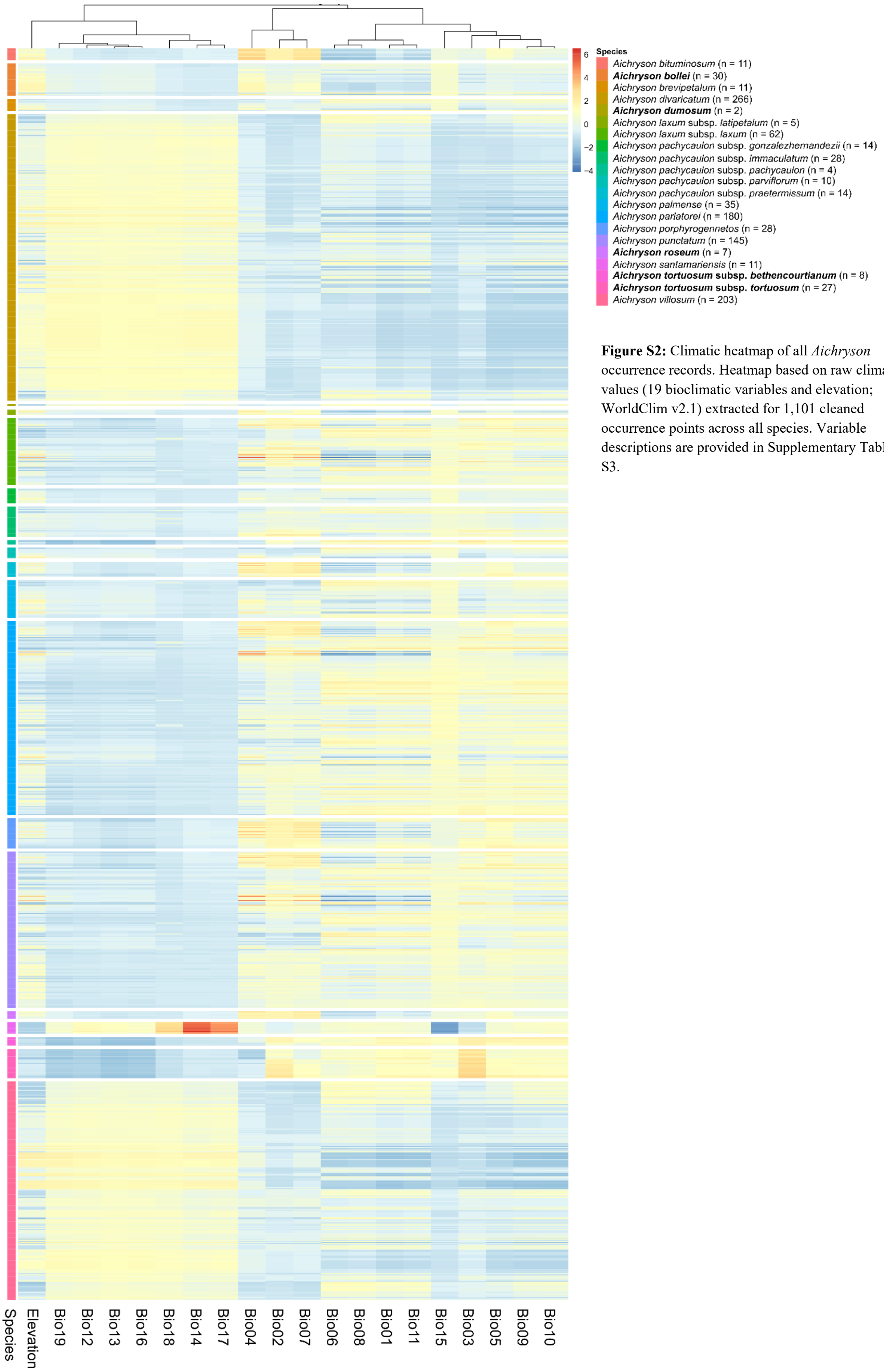

Figure S2: Climatic heatmap of all *Aichryson* occurrence records. Heatmap based on raw climatic values (19 bioclimatic variables and elevation; WorldClim v2.1) extracted for 1,101 cleaned occurrence points across all species. Variable descriptions are provided in Supplementary Table S3.

Supplementary Figure 3

Boxplots for each bioclimatic variable per species. Variable descriptions are provided in Supplementary Table S3

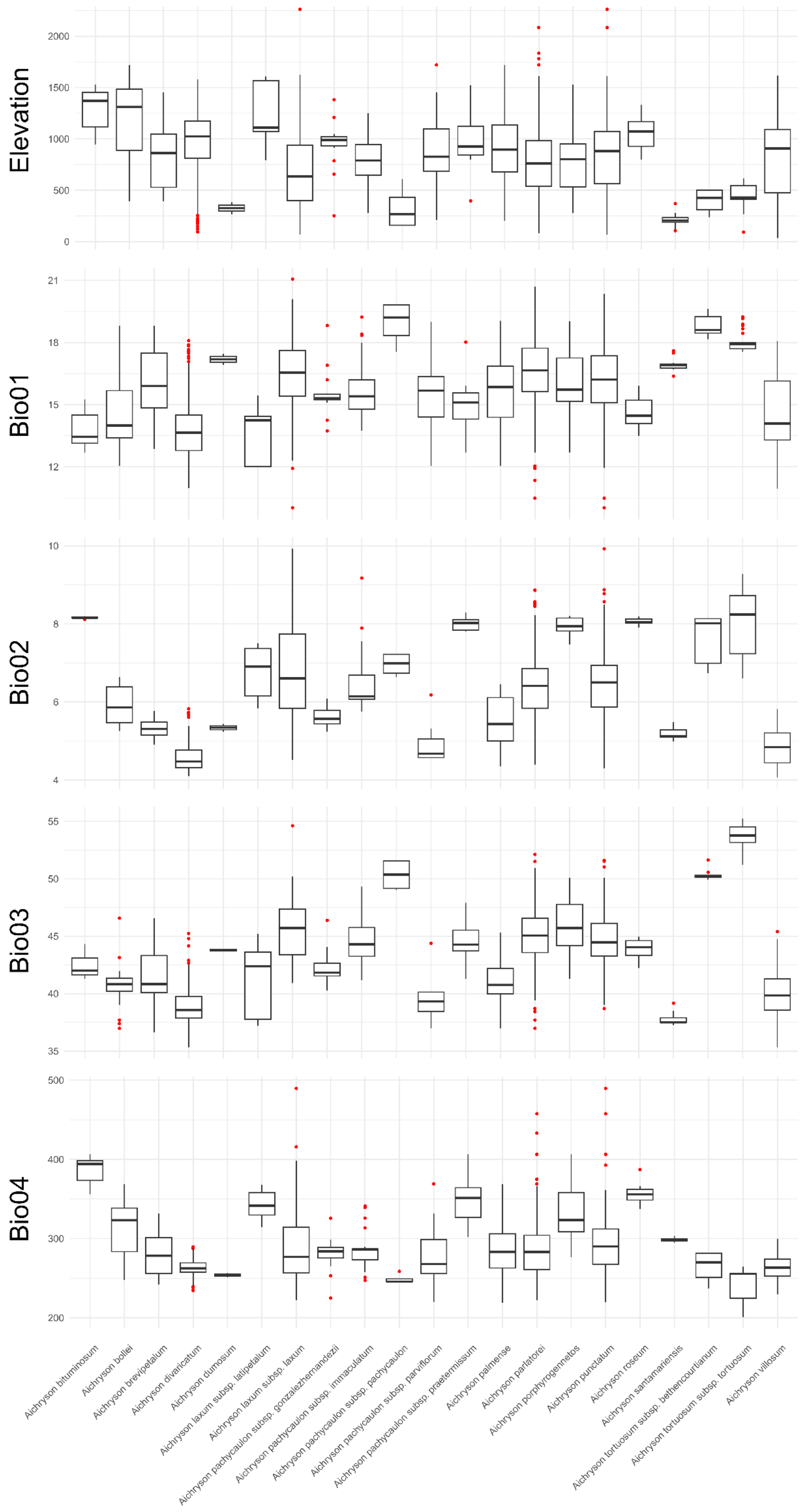

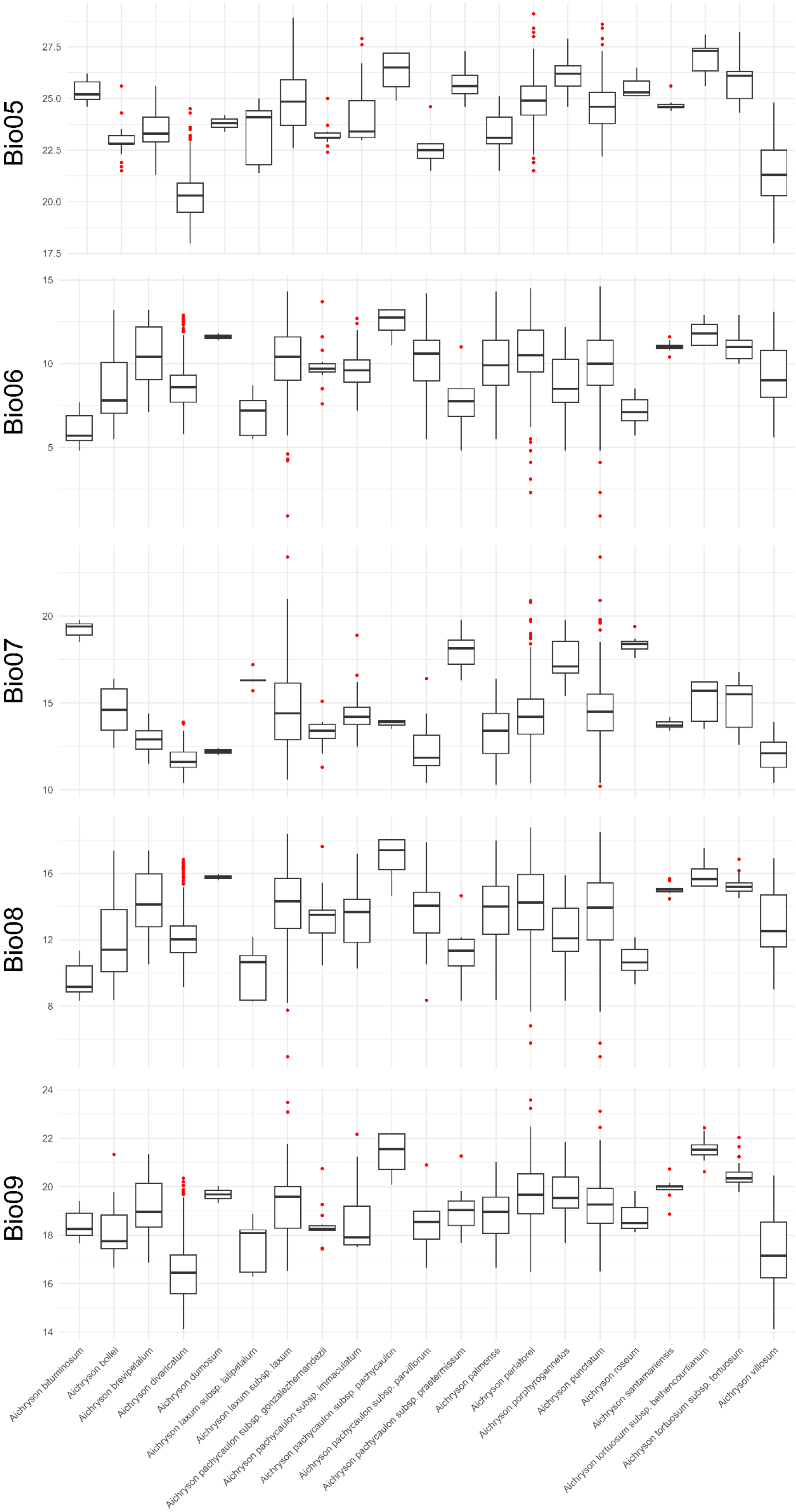
Bio05
27.5
25.0
22.5
20.0
17.5
Bio06
15
10
5
Bio07
20
15
10
Bio08
16
12
8
Bio09
24
22
20
18
16
14
Aichryson bituminosum
Aichryson bollei
Aichryson brevipetalum
Aichryson divaricatum
Aichryson dumosum
Aichryson laxum subsp. latipetalum
Aichryson laxum subsp. laxum
Aichryson pachycaulon subsp. gonzalezhernandezii
Aichryson pachycaulon subsp. immaculatum
Aichryson pachycaulon subsp. pachycaulon
Aichryson pachycaulon subsp. parviflorum
Aichryson pachycaulon subsp. praetermissum
Aichryson palmense
Aichryson parlatorei
Aichryson porphyrogennetos
Aichryson punctatum
Aichryson roseum
Aichryson santamariensis
Aichryson tortuosum subsp. bethencourtianum
Aichryson tortuosum subsp. tortuosum
Aichryson villosum

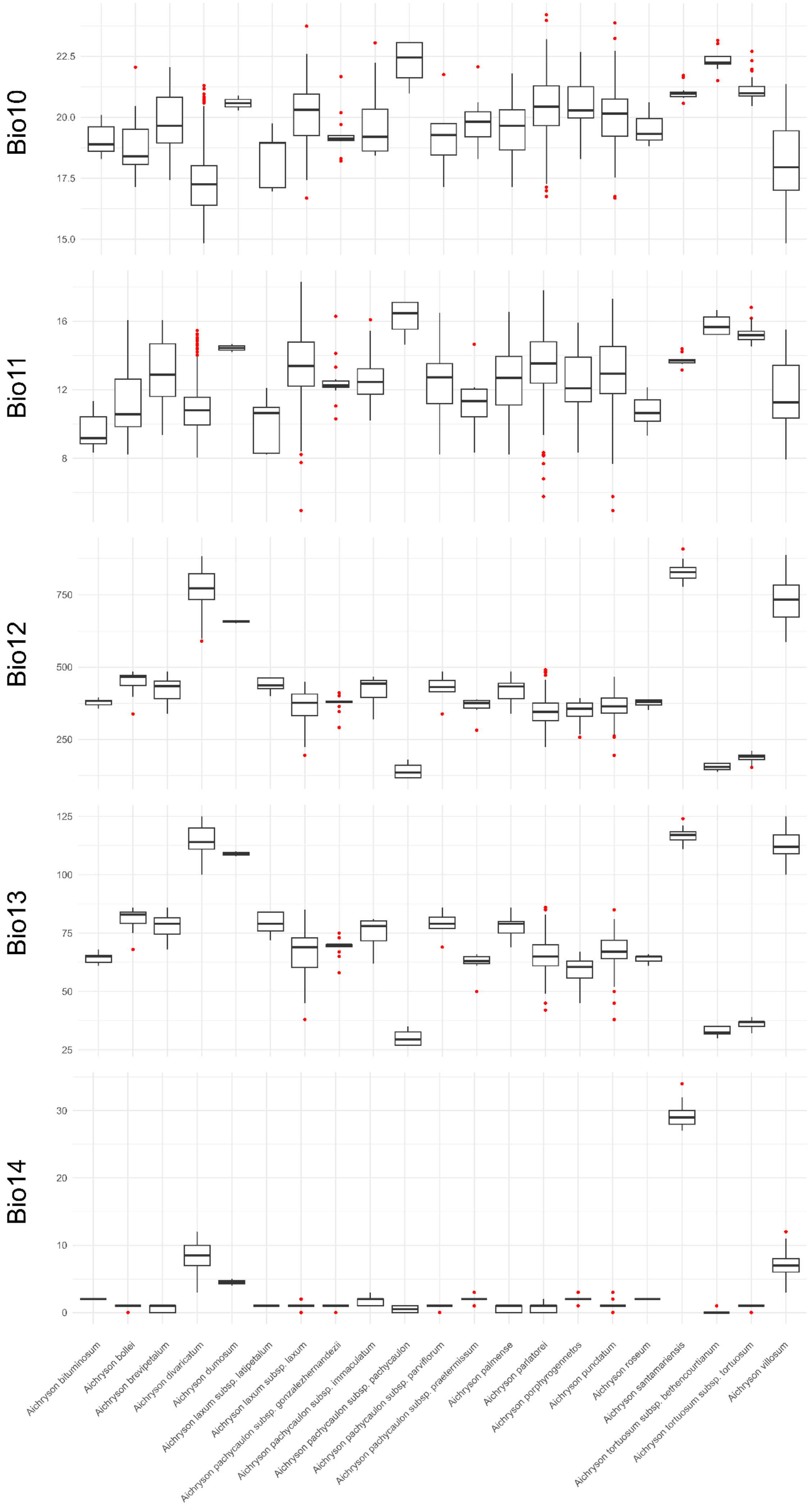
Bio10
15.0
17.5
20.0
22.5
Bio11
8
12
16
Bio12
250
500
750
Bio13
25
50
75
100
125
Bio14
0
10
20
30
Aichryson bituminosum
Aichryson bollei
Aichryson brevipetalum
Aichryson divaricatum
Aichryson dumosum
Aichryson laxum subsp. latipetalum
Aichryson laxum subsp. laxum
Aichryson pachycaulon subsp. gonzalezhernandezii
Aichryson pachycaulon subsp. immaculatum
Aichryson pachycaulon subsp. pachycaulon
Aichryson pachycaulon subsp. parviflorum
Aichryson pachycaulon subsp. praetermissum
Aichryson palmense
Aichryson parlatorei
Aichryson porphyrogennetos
Aichryson punctatum
Aichryson roseum
Aichryson santamariensis
Aichryson tortuosum subsp. bethencourtianum
Aichryson tortuosum subsp. tortuosum
Aichryson villosum

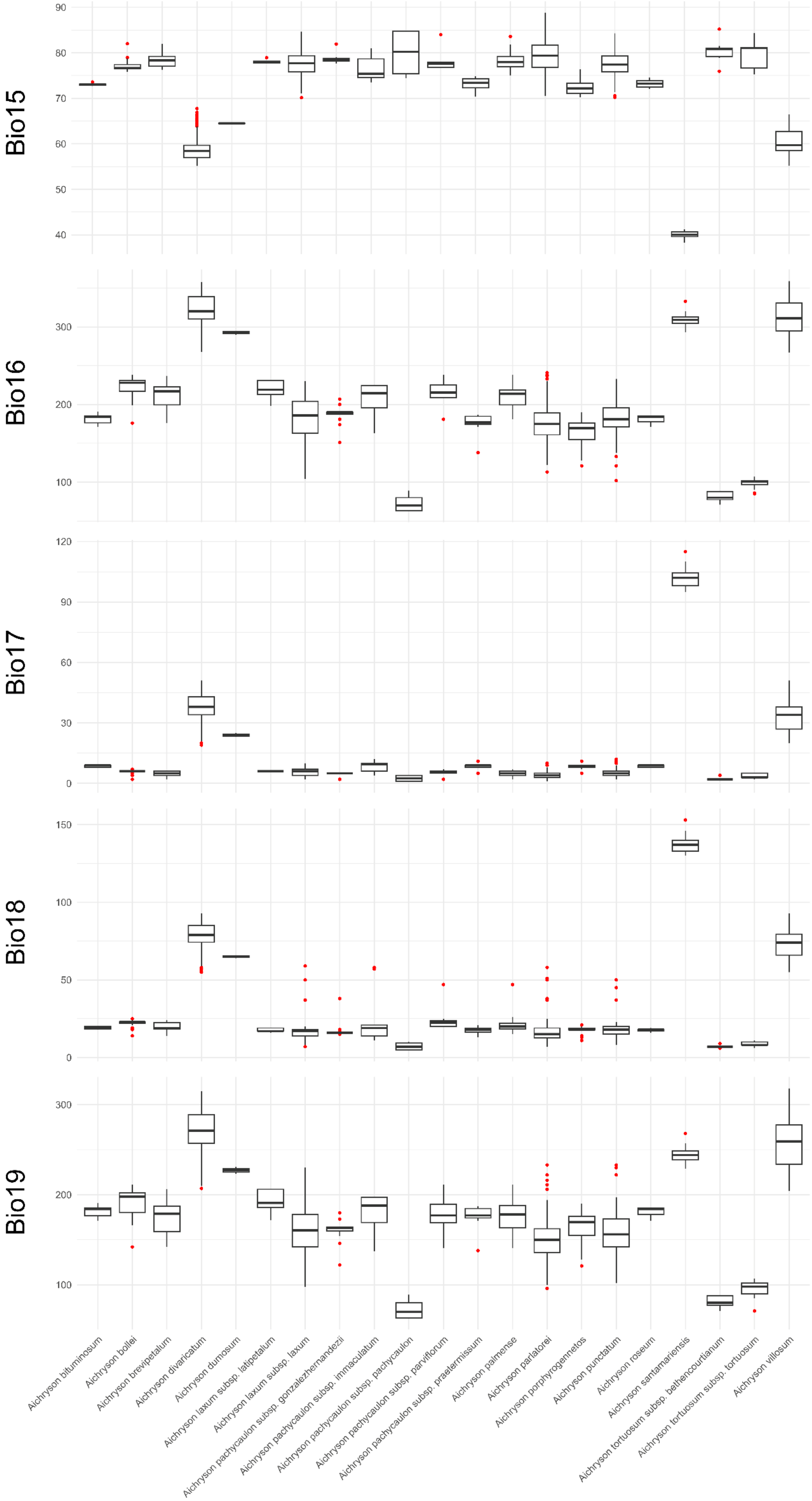
Bio15
Bio16
Bio17
Bio18
Bio19
90
80
70
60
50
40
300
200
100
120
90
60
30
0
150
100
50
0
300
200
100
Aichryson bituminosum
Aichryson bollei
Aichryson brevipetalum
Aichryson divaricatum
Aichryson dumosum
Aichryson laxum subsp. latipetalum
Aichryson laxum subsp. laxum
Aichryson pachycaulon subsp. gonzalezhernandezii
Aichryson pachycaulon subsp. immaculatum
Aichryson pachycaulon subsp. pachycaulon
Aichryson pachycaulon subsp. parviflorum
Aichryson pachycaulon subsp. praetermissum
Aichryson palmense
Aichryson parlatorei
Aichryson porphyrogennetos
Aichryson punctatum
Aichryson roseum
Aichryson santamariensis
Aichryson tortuosum subsp. bethencourtianum
Aichryson tortuosum subsp. tortuosum
Aichryson villosum
