# Supplementary material for: Evolutionary Changes in Crassulacean Acid Metabolism (CAM) and Related Traits During the Diversification of Aichryson (Crassulaceae) on the Macaronesian Islands
Source: Ecol Evol. 2026 Jan 2;16(1):e72864. doi: 10.1002/ece3.72864 (PMC12758979; doi:10.1002/ece3.72864)
Supplement: Supplementary file 4 — Table S3: (i) Description of the environmental variables used in this study, (ii) PCA loadings for all 19 bioclimatic variables and elevation describing the climatic niche space of Aichryson across the Macaronesian archipelagos (Figure 1), (iii) PCA loadings for all 19 bioclimatic variables and elevation based on species‐level means (Figure 2). [file ECE3-16-e72864-s003.pdf]

# Supplementary Table S3

i. The environmental variables used in this study. For the complete description of each variable, please refer to the data source, WorldClim v2.1 (Fick & Hijmans, 2017).

| ***Variable*** | ***Description*** | ***Unit*** |
|---|---|---|
| *Bio01* | Mean annual air temperature. | °C |
| *Bio02* | Mean diurnal air temperature range. (Mean of monthly (max temp - min temp)) | °C |
| *Bio03* | Isothermality | % |
| *Bio04* | Temperature seasonality | °C |
| *Bio05* | Maximum temperature of the warmest month. | °C |
| *Bio06* | Minimum temperature of the coldest month. | °C |
| *Bio07* | Temperature annual range: The difference between the maximum temperature of the warmest month and the minimum temperature of the coldest month (bio5-bio6). | °C |
| *Bio08* | Mean temperature of the wettest quarter. | °C |
| *Bio09* | Mean temperature of the driest quarter. | °C |
| *Bio10* | Mean temperature of the warmest quarter. | °C |
| *Bio11* | Mean temperature of the coldest quarter. | °C |
| *Bio12* | Annual precipitation. | mm |
| *Bio13* | Precipitation amount of the wettest month. | mm |
| *Bio14* | Precipitation of the driest month. | mm |
| *Bio15* | Precipitation seasonality: The coefficient of variation of monthly or seasonal precipitation. | % |
| *Bio16* | Precipitation of the wettest quarter. | mm |
| *Bio17* | Precipitation of the driest quarter. | mm |
| *Bio18* | Precipitation of the warmest quarter. | mm |
| *Bio19* | Precipitation of the coldest quarter. | mm |
| *Elevation* | Topographic elevation. | m |

ii. PCA loadings for all 19 bioclimatic variables and elevation describing the climatic niche space of *Aichryson* across the Macaronesian archipelagos (Figure 1).

| ***Variable*** | ***PC1*** | ***PC2*** |
|---|---|---|
| *Bio01* | 0.23818195 | -0.21592608 |
| *Bio02* | 0.18260983 | 0.28441216 |
| *Bio03* | 0.24600453 | 0.0130721 |
| *Bio04* | 0.00810858 | 0.41703087 |
| *Bio05* | 0.25908424 | 0.05549079 |
| *Bio06* | 0.17957756 | -0.3369399 |
| *Bio07* | 0.109978 | 0.37927036 |
| *Bio08* | 0.18114619 | -0.33345081 |
| *Bio09* | 0.25875292 | -0.1002512 |
| *Bio10* | 0.25707794 | -0.11491066 |
| *Bio11* | 0.21748908 | -0.27262958 |
| *Bio12* | -0.26217877 | -0.11061402 |
| *Bio13* | -0.25720347 | -0.12040628 |
| *Bio14* | -0.22333786 | -0.12446051 |
| *Bio15* | 0.24169485 | 0.12536652 |
| *Bio16* | -0.26185055 | -0.10164999 |
| *Bio17* | -0.23553639 | -0.13751546 |
| *Bio18* | -0.243462 | -0.16383563 |
| *Bio19* | -0.26866158 | -0.03704499 |
| *Elevation* | -0.16133852 | 0.33730878 |

iii. PCA loadings for all 19 bioclimatic variables and elevation based on species-level means (Figure 2).

| ***Variable*** | ***PC1*** | ***PC2*** |
|---|---|---|
| *Bio01* | 0.24135448 | -0.24791566 |
| *Bio02* | 0.19984724 | 0.20605331 |
| *Bio03* | 0.28872009 | -0.00873142 |
| *Bio04* | -0.06588717 | 0.30031308 |
| *Bio05* | 0.25307149 | 0.03312143 |
| *Bio06* | 0.14633266 | -0.32761866 |
| *Bio07* | 0.08449934 | 0.27892937 |
| *Bio08* | 0.15510914 | -0.32956046 |
| *Bio09* | 0.26833164 | -0.15079992 |
| *Bio10* | 0.25913277 | -0.16942466 |
| *Bio11* | 0.21528483 | -0.2818703 |
| *Bio12* | -0.28626556 | -0.14654299 |
| *Bio13* | -0.29075988 | -0.12530817 |
| *Bio14* | -0.16111419 | -0.23244599 |
| *Bio15* | 0.20696166 | 0.20622393 |
| *Bio16* | -0.29671051 | -0.10271783 |
| *Bio17* | -0.178585 | -0.23909478 |
| *Bio18* | -0.21678798 | -0.24568752 |
| *Bio19* | -0.3051331 | -0.04006509 |
| *Elevation* | -0.1289919 | 0.34154869 |
